# miRNAs-neutrophil axis: novel insights into acute lung injury and chronic inflammatory lung diseases

**DOI:** 10.3389/fimmu.2026.1803762

**Published:** 2026-04-13

**Authors:** Xingning Lai, Jie Zhong, Chenxi Liao, Baojiang Chen, Wensheng Zhang, Ren Liao

**Affiliations:** 1 Department of Anesthesiology, West China Hospital, Sichuan University, Chengdu, China; 2 Laboratory of Anesthesia and Critical Care Medicine, National-Local Joint Engineering Research Center of Translational Medicine of Anesthesiology, West China Hospital, Sichuan University, Chengdu, China

**Keywords:** acute lung injury, chronic inflammatory lung diseases, inflammation, miRNA, neutrophil, neutrophil extracellular trap

## Abstract

Acute lung injury (ALI) and chronic inflammatory lung disorders constitute significant clinical burdens with high morbidity and mortality. Neutrophils are commonly found in lung diseases and serve as pivotal effector cells governing pulmonary inflammation. MicroRNAs (miRNAs) have emerged as critical regulators of neutrophil biology and inflammatory responses. This review summarizes current understanding of miRNA-mediated regulation of neutrophil dynamics, including granulopoiesis, recruitment, and neutrophil extracellular trap (NET) formation, and delineates the contributions of the miRNA-neutrophil axis to the pathogenesis of ALI, asthma, pulmonary fibrosis, and lung cancer. This review describes the dual roles of miRNAs, acting either as promoters or suppressors in neutrophil biology and inflammatory lung diseases, and highlights mechanistic insights into miRNA-mediated pathways influencing neutrophil-driven inflammation and tissue injury. Furthermore, we explore the therapy potential of targeting the miRNA-neutrophil axis, evaluating miRNA-based therapeutics as novel strategies to modulate neutrophil-driven pathology in lung diseases. By elucidating the miRNAs-neutrophil axis as an integrative conceptual framework, this review aims to spotlight promising avenues for developing therapies for debilitating acute and chronic respiratory disorders.

## 1 Introduction

Inflammatory lung diseases (including acute lung injury (ALI) and chronic lung diseases) are considered to be the leading cause of high morbidity and mortality worldwide (1). Many patients need positive-pressure ventilation to maintain adequate oxygenation because there are no specific therapeutic strategies for inflammatory lung diseases. Accumulated evidence suggests that the excessive infiltration of neutrophils represents a hallmark of inflammatory lung diseases (2). Neutrophils account for 40% to 70% of all circulating white blood cells in the human blood and are a critical line of defense for the innate immune system. They play an important role in host defense in response to invading pathogens in the lungs. On the other hand, they can also cause tissue damage when their response is excessive or dysregulated (3). Neutrophils quickly mobilize to the infection or injury sites to perform critical defense functions through phagocytosis, intracellular degradation, reactive oxygen species (ROS) production, and the formation of neutrophil extracellular traps (NETs) (4). However, uncontrolled activation of neutrophils leads to damage to the alveolar epithelium and endothelial barrier as well as increases vascular permeability. Delayed apoptosis of neutrophils and impaired clearance of macrophages contribute to the release of intracellular contents, further exacerbating inflammation and recruiting more immune cells (5).

MicroRNAs (miRNAs) are small single-stranded RNAs with a length of 22 nucleotides. miRNA biogenesis begins with transcription into primary miRNAs (pri-miRNAs), which are processed in the nucleus by the Drosha and DGCR8 into precursor miRNAs (pre-miRNAs). After export to the cytoplasm, pre-miRNAs are cleaved by Dicer and TAR RNA binding protein (TRBP) to generate mature miRNA duplexes, which are incorporated into the RNA-induced silencing complex (RISC) (6). miRNAs bind to the 3’untranslated region (3’UTR) of messenger RNA (mRNA) and repress gene expression by suppressing mRNA translation or degrading mRNA. Each miRNA can target various mRNAs, thereby mediating the expression of several genes. Each gene can also be regulated by multiple miRNAs. Functionally, miRNAs can regulate various cellular processes, such as cell proliferation, differentiation, and apoptosis (7). Dysregulation of miRNAs is implicated in various diseases, including cancer, autoimmune disorders, viral infections, and cardiovascular diseases (8, 9). MiRNA profiles in tissues, blood, or other body fluids (circulating miRNAs) serve as sensitive, non-invasive indicators for early disease detection (e.g., specific cancer types), classification, and outcome prediction (10).

Numerous reviews mainly focus on the roles of miRNAs in inflammatory lung diseases (11, 12) or the contribution of neutrophils to pulmonary inflammation (13, 14). These studies have established essential foundations, identifying individual miRNAs dysregulated in specific disease contexts and characterizing neutrophil functions in host defense and tissue injury. However, the comprehensive synthesis of the cross-talk between miRNA and neutrophil biology in various lung pathologies is not yet fully understood. Rather than focusing on the regulatory roles of individual miRNAs in specific lung diseases or on neutrophil effects in isolation, the present review introduces a mechanistic framework centered on the miRNA-neutrophil axis. This review collects current studies that examine miRNAs as critical modulators to control neutrophil dynamics (e.g., granulopoiesis, recruitment, and NET formation) and how these regulatory interactions influence disease pathogenesis across ALI, asthma, pulmonary fibrosis, and lung cancer. This article describes a mechanistic understanding of how the miRNA-neutrophil axis drives both protective and pathological outcomes. This framework not only advances fundamental knowledge of neutrophil biology but also provides a clue for developing targeted interventions.

## 2 miRNA regulation of neutrophil biology

### 2.1 Granulopoiesis

#### 2.1.1 The positive roles of miRNAs in granulopoiesis

Granulopoiesis is a process during which neutrophils are generated and obtain functional properties in bone marrow niches. Myeloid progenitors undergo a process called emergency granulopoiesis to significantly increase the number of neutrophils, which can adapt to the high demand for neutrophils after infection or injury (15). One study found that miR-223 is highly expressed in myeloid cells and absent in lymphocytes. It can recognize the 3’UTR of nuclear factor 1 A-type (NFI-A) and represses the translation of NFI-A mRNA. NFI-A binds to the miR-223 promoter and impedes miR-223 expression, which can inhibit granulocytic differentiation. CCAAT enhancer protein alpha (C/EBPα) is activated to replace NFI-A after retinoic acid treatment and enhances miR-223 expression, finally stimulating granulocytic differentiation of myeloid precursors (16). Vian et al. indicated that miR-223 up-regulation stimulates granulopoiesis and dampens erythroid and monocytic differentiation (17). miR-34a expression is reported to be induced by C/EBPα during granulopoiesis. Increased miR-223 and miR-34a promote granulocytic differentiation via targeting E2F1 and E2F3, respectively (18, 19). Trib2 is shown to restrain C/EBPα function, and its expression can be upregulated by Notch1. The miR-30c level is raised via C/EBPα during granulopoiesis, and increased miR-30c can impede Notch1 and Trib2 to boost granulocytic differentiation (20). miR-125a has been implicated in the pathogenesis of various pathological conditions, including systemic lupus erythematosus and ischemic stroke, and has been identified as a prognostic indicator of human cancer (21). Qin et al. reported that inhibition of suppressor of cytokine signaling 3 (SOCS3) increases the number of neutrophils in the bone marrow without showing measurable effects on granulocytic progenitors. Genetic deletion of miR-125a lessens cell proliferation of immature neutrophils mediated by granulocyte colony-stimulating factor (G-CSF) during granulopoiesis, an effect mediated through its targeted regulation of SOCS3 (22). miR-129 biogenesis can be enhanced by KH-type splicing regulatory protein (KSRP) during granulopoiesis. Increased miR-129 can stimulate granulocyte differentiation via targeting runt-related transcription factor 1 (RUNX1) (23).

#### 2.1.2 The negative roles of miRNAs in granulopoiesis

miR-130a expression is significantly high in proliferating granulocytic precursors, and its expression decreases as the cells mature. Overexpressed miR-130a decreases Smad4 expression to reduce the sensitivity of cells to transforming growth factor beta 1 (TGF-β1) stimulation. As a result, miR-130a overexpression facilitates cell proliferation of granulocytic precursors and represses granulocytic differentiation (24). Another study found that low expression of miR-130a causes high levels of C/EBPϵ and increases mRNA expression of granule proteins (lactoferrin and cathelicidin antimicrobial protein) to promote cell maturation of immature neutrophil precursors, which is beneficial for granulopoiesis (25). C/EBPα binds to the miR-182 promoter and restrains miR-182 expression during myeloid differentiation. Enforced miR-182 expression can decline C/EBPα levels and impair granulopoiesis. The interaction between miR-182 and C/EBPα plays a key regulatory role in granulocyte generation, and damaging the miR-182-C/EBPα balance impairs granulocyte differentiation (26). Normal granulocytic differentiation depends on the zinc finger protein growth factor independent-1 (GFI-1). miR-196b and miR-21expression are facilitated by GFI-1 deficiency, which can restrain G-CSF-stimulated granulopoiesis (27). Another study indicated that miR-223 deletion leads to enhanced proliferation of the granulocyte progenitors through targeting myocyte-specific enhancer factor 2C (MEF2C) (28).

### 2.2 Neutrophil recruitment

#### 2.2.1 The positive roles of miRNAs in neutrophil recruitment

The recruitment of neutrophils to sites of tissue injury or infection is first driven by the chemotactic guidance of inflammatory mediators, followed by firm attachment to the endothelial cells and subsequent transendothelial migration (29, 30). miR-142-3p is a hematopoietic-specific miRNA and is highly enriched in zebrafish embryos and definitive hematopoietic organs. miR-142-3p knockout significantly decreases the number of neutrophils during the embryonic period and impairs neutrophil chemotactic migration under inflammatory conditions via inducing an abnormal activation of the interferon γ (IFN-γ) pathway (31). Extracellular vesicles (EVs) are membranous particles produced by cells into the extracellular space, and EVs contain surface molecules together with the bioactive cargos to affect recipient cells. Exosomes (30 to 150 nm in diameter), microvesicles (100 to 1000 nm in diameter), and apoptotic bodies (100 to 5000 nm in diameter) are the three subtypes of EVs that are separated according to size range and biogenesis (32). Exosomes are generated from the endocytic pathway involved in plasma membrane budding and the formation of multivesicular endosomes containing intraluminal vesicles. Exosomes govern cell-cell communication by transporting bioactive cargoes (e.g., proteins, lipids, and nucleic acids) from donor cells to recipient cells. Exosomes contain a lipid bilayer membrane that protects against enzymatic degradation of these intracellular contents (33). miR-126 is enriched in endothelial cell-derived EVs expressing vascular cell adhesion molecule-1 (VCAM-1) under the stimulation of tumor necrosis factor-α (TNF-α). EVs expressing miR-126 and VCAM-1 can repress the expression of retention chemokines and raise the mobilization signal, which can promote neutrophil mobilization from the spleen to peripheral blood (34). EV miR-143-3p secreted from astrocytes can increase miR-143-3p levels in the recipient endothelial cells. miR-143-3p blocks lysosomal hydrolysis ability and represses the autophagic degradation of cell adhesion molecules through inhibiting ATP6V1A. Enhanced expression of cell adhesion molecules can promote neutrophil adhesion to endothelial cells and neutrophil transendothelial cell migration into the brain (35). miR-1287-5p is abundant in plasma exosomes collected from patients with microscopic polyangiitis. Exosomal miR-1287-5p can facilitate adhesion molecules (including intercellular adhesion molecule-1 (ICAM-1) and E-selection) in endothelial cells. Exosomal miR-1287-5p enhances neutrophil adhesion to endothelial cells via suppressing casitas B-lineage lymphoma (CBL) (36).

#### 2.2.2 The negative roles of miRNAs in neutrophil recruitment

Hsu et al. found several miRNAs that can suppress neutrophil recruitment by performing an *in vivo* miRNA screen in zebrafish and demonstrated that miR-199–3 overexpression leads to the strongest inhibition of neutrophil activity. This study also suggested that overexpression of miR-199–3 can dampen neutrophil chemotaxis and migration via targeting cyclin-dependent kinase 2 (CDK2). miR-199–3 up-regulation or CDK2 repression elevates survival rates of zebrafish undergoing bacterial infection or sterile inflammation (37). Hsu et al. also revealed that up-regulated miR-722 can suppress neutrophil motility and neutrophil recruitment to injury or infection via targeting Rac2 in zebrafish. On the other hand, miR-722 mimic protects zebrafish from sterile systemic inflammation and also elevates survival rates *in vivo* (38). Subsequent research indicated that increasing zebrafish miR-722 damages cell cytoskeleton remodeling and abrogates cell migration in human neutrophil cells. In addition, elevating miR-722 diminishes ROS generation, as evidenced by decreased production of hydrogen peroxide in neutrophils (39). Consistent with this, Liu et al. also showed that miR-485-5p up-regulation reduces ROS production and neutrophil cell adhesion to endothelial cells via suppressing ICAM-1 (40). miR-375 is neutrophil-specific and shows no impact on neutrophil biogenesis or neutrophil survival in zebrafish. Increased miR-375 can repress neutrophil chemotaxis and migration via inhibiting Cathepsin B (CtsB) in response to bacterial infection and tail transection (41). Zhou et al. displayed that miR-223 represses the nuclear factor-kappaB (NF-κB) pathway by targeting genes *Cul1a*, *Cul1b*, *Traf6*, and *Tab1*. miR-223 deficiency can enhance neutrophil recruitment toward the injured sites and promote neutrophilic inflammation via the activation of the NF-κB pathway *in vivo* (42). miR-146a acts as a critical regulatory factor for innate immunity in keratinocytes, and its expression can be induced by toll-like receptor 2 (TLR2) stimulation through the NF-κB and mitogen-activated protein kinase (MAPK) pathways. miR-146a overexpression in keratinocytes significantly impedes the secretion of inflammatory mediators (interleukin-8 (IL-8), C-C motif chemokine ligand 20 (CCL20), and TNF-α) to decrease chemotactic migration of neutrophils (43). Kivihall et al. showed that miR-146a mimics in bronchial epithelial cells elevate miR-146a levels and high expression of miR-146a suppresses the production of IL-8 and C-X-C motif chemokine ligand 1 (CXCL1) to reduce neutrophil migration (44). miR-451 is abundant in red blood cells, platelets, and neutrophils. miR-451 overexpression inhibits neutrophil migration via repressing the p38 MAPK pathway through targeting Rab5a (45). Similarly, elevated miR-328-3p restrains the NF-κB pathway to repress IL-1β and IL-18 secretion of human umbilical vein endothelial cells (HUVECs) treated with oxygen-glucose deprivation. Increased miR-328-3p finally attenuates neutrophil migration (46).

### 2.3 Neutrophil extracellular trap formation

#### 2.3.1 The positive roles of miRNAs in neutrophil extracellular trap formation

NETs, which were first found by Brinkmann et al. in 2004, are extracellular fibrous network structures produced by activated neutrophils under the stimulation of pathogens, immunological stimuli, tumor-associated stimuli, phorbol 12-myristate 13-acetate (PMA), or lipopolysaccharide (LPS) (47). NET formation is associated with two different pathways, NETosis (a unique cell death process that is distinct from cell necrosis and cell apoptosis) and non-lytic NETosis (a process that occurs without cell death). NETs contain several kinds of components, such as DNA, histones, neutrophil elastase, matrix metalloproteinases (MMPs), cathepsins, chemokines, and cytokines (48). NETs participate in host defense against pathogenic invasion, immune regulation that involves various cells (e.g., macrophages, dendritic cells, natural killer cells, B cells, and T cells), immunothrombosis, and wound healing under normal physiological conditions. However, exaggerated NETs contribute to infectious diseases, autoimmune disorders, metabolic dysregulation, thrombosis, and malignant cancers (49). miR-155 expression and mRNA levels of peptidylarginine deiminase 4 (PAD4) are enhanced via PMA in neutrophils isolated from bone marrow. Increased miR-155 can interact with the AU-rich elements of the PAD4 3′-UTR region and raise PAD4 mRNA levels in neutrophils, resulting in promoting NET formation upon PMA (50). Knockdown of miR-155 abolishes sepsis-induced NET formation via diminishing plasma DNA-histone complexes and citrullinated histone 3 (H3Cit) through PAD4. Moreover, eliminating miR-155 decreases lung and circulating levels of CXCL1 and CXCL2, which can decline neutrophil recruitment into the mouse lung *in vivo* (51). Both miR-15b-5p and miR-378a-3p are highly expressed in platelet-derived exosomes and directly target phosphoinositide-dependent protein kinase 1 (PDK1). Exosomal miR-15b-5p and miR-378a-3p enhance NET formation via repressing the protein kinase B (AKT)/mammalian target of rapamycin (mTOR) pathway through PDK1 (52). Wang et al. indicated that miR-142a-3p is expressed in liver-derived EVs. EV miR-142a-3p potentiates NET formation via restraining IL-10 expression through inhibiting WASL (53). miR-146a-5p is enriched in macrophage-derived exosomes stimulated by oxidized low-density lipoprotein (ox-LDL) and can be transferred into neutrophils. Exosomal miR-146a-5p directly suppresses polynucleotide kinase 3’−phosphatase (PNKP) expression, thereby reducing the interaction between PNKP and dolichyl-diphosphooligosaccharide-protein glycosyltransferase non-catalytic subunit (DDOST). This leads to enhanced phosphorylation of DDOST and subsequent up-regulation of junctional adhesion molecule-like protein (JAGN1). Exosomal miR-146a-5p ultimately facilitates ROS production and NET formation through JAGN1 (54). miR-505 expression is up-regulated in exosomes isolated from ox-LDL-treated HUVECs. Exosomal miR-505 abrogates sirtuin 3 (SIRT3) expression in neutrophils and thereby elicits NET formation (55).

#### 2.3.2 The negative roles of miRNAs in neutrophil extracellular trap formation

Ye et al. revealed that miR-223 deletion can encourage neutrophil elastase production to accelerate NET formation, resulting in eliciting oxidative stress and neutrophilic inflammation (56). It is demonstrated that declined miR-146a levels induced by IL-6 in alveolar cells can promote high-mobility group box 1 (HMGB1) release, which can attract neutrophils expressing TLR4 to trigger pulmonary NET formation (57). miR-16-5p can be induced and reduced by hydrogen sulfide and PMA in neutrophils, respectively. Up-regulated miR-16-5p attenuates phosphatidylinositol 3-kinase (PI3K)/AKT and extracellular signal-regulated kinase (ERK) pathways through hindering PIK3R1 and rapidly accelerated fibrosarcoma-1 (RAF-1), respectively. Elevated miR-16-5p ultimately reduces NET formation via abrogating calcium ion outflow, ROS generation, and autophagy (58). Yang et al. demonstrated that overexpressed miR-1696 inhibits the activation of MAPK (p38, ERK, and c-Jun N-terminal kinase (JNK)) and PI3K/AKT pathways via down-regulating its downstream target glutathione peroxidase 3 (GPX3), which can abolish NET formation via diminishing the expression of myeloperoxidase (MPO) and protein kinase C (PKC) (59). Yang et al. proved that decreased miR-4512 in monocytes and macrophages facilitates the secretion of inflammatory cytokines (IL-1β, IL-12, TNF-α, IFN-γ, and IL-17) and restrains IL-4 and IL-10 release. The supernatants isolated from monocytes and macrophages transfected with agomiR-4512 can reduce NETosis, as evidenced by diminished MPO, neutrophil elastase, and cell-free DNA (60). miR-26b-5p can be delivered into neutrophils through exosomes derived from platelet-rich plasma. Exosomal miR-26b-5p suppresses NETs via reducing MMP-8 (61). Consistent evidence shows that miR-5119 and miR-5106 inhibit NET formation via targeting programmed death-ligand 1 (PD-L1) (62) and Slc39a2 (63), respectively.

**Figure 1:**
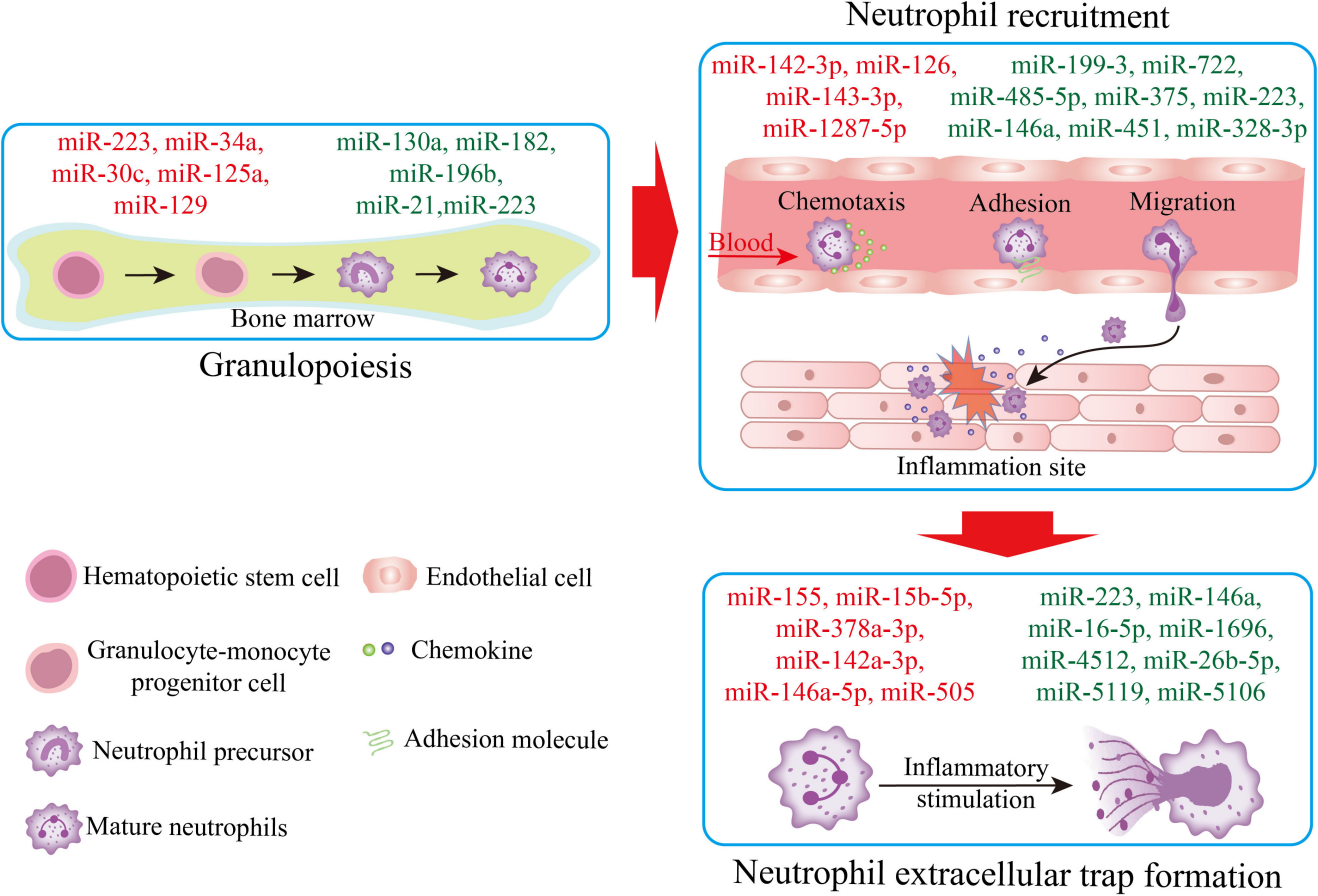
miRNA regulation across neutrophil life stages. The schematic illustrates three key phases of neutrophil biology: granulopoiesis in the bone marrow, recruitment to inflammation sites, and NET formation in the lung microenvironment. Related miRNAs are represented by different colors: miRNAs in red indicate promoters, miRNAs in green indicate inhibitors.

As mentioned above, miRNAs play positive or negative roles in granulopoiesis, neutrophil recruitment, and NET formation in response to certain conditions (**Figure 1**, **Table 1**). miRNAs regulate their targeted mRNAs to influence the activation of signal pathways to affect neutrophil biology. During granulopoiesis, miRNA-mRNA networks mainly influence granulocyte differentiation (**Table 1**). In neutrophil recruitment, miRNA-mRNA networks regulate neutrophil chemotaxis, migration, and adhesion to endothelial cells (**Table 1**). In NET formation, miRNA-mRNA networks modulate NET-associated factors, including MPO and neutrophil elastase. The dysregulated miRNA-mRNA-pathway axis can lead to dysfunctional neutrophils.

**Table 1:** Summary of miRNA effects on neutrophil biology.

Process	miRNA	Key target(s)/pathway	Outcome	Role	Reference
Granulopoiesis	miR-223	C/EBPα, NFI-A, E2F1	Differentiation	Promoter	(16, 18)
	miR-34a	E2F3	Differentiation	Promoter	(19)
	miR-30c	Notch1, Trib2	Differentiation	Promoter	(20)
	miR-125a	SOCS3	Proliferation	Promoter	(22)
	miR-129	RUNX1	Differentiation	Promoter	(23)
	miR-130a	Smad4	Differentiation	Inhibitor	(24)
		C/EBPϵ	Differentiation	Inhibitor	(25)
	miR-182	C/EBPα	Differentiation	Inhibitor	(26)
	miR-196b, miR-21	GFI1	Differentiation	Inhibitor	(27)
	miR-223	MEF2C	Proliferation	Inhibitor	(28)
Neutrophil recruitment	miR-142-3p	IFN-γ	Migration	Promoter	(31)
	miR-143-3p	ATP6V1A	Adhesion, migration	Promoter	(35)
	miR-1287-5p	CBL	Adhesion	Promoter	(36)
	miR-199-3	CDK2	Chemotaxis, migration	Inhibitor	(37)
	miR-722	Rac2	Motility, recruitment	Inhibitor	(38)
	miR-485-5p	ICAM-1	Adhesion	Inhibitor	(40)
	miR-375	CtsB	Chemotaxis, migration	Inhibitor	(41)
	miR-223	NF-κB	Recruitment	Inhibitor	(42)
	miR-146a	NF-κB, MAPK	Migration	Inhibitor	(43)
	miR-451	Rab5a, p38 MAPK	Migration	Inhibitor	(45)
	miR-328-3p	NF-κB	Migration	Inhibitor	(46)
NETs	miR-155	PAD4	NET formation	Promoter	(50)
	miR-15b-5p, miR-378a-3p	PDK1→AKT/mTOR	NET formation	Promoter	(52)
	miR-142a-3p	WASL→IL-10	NET formation	Promoter	(53)
	miR-146a-5p	JAGN1	NET formation	Promoter	(54)
	miR-505	SIRT3	NET formation	Promoter	(55)
	miR-223	Neutrophil elastase	NET formation	Inhibitor	(56)
	miR-146a	HMGB1	NET formation	Inhibitor	(57)
	miR-16-5p	PIK3R1, RAF-1→PI3K/AKT, ERK	NET formation	Inhibitor	(58)
	miR-1696	GPX3→MAPK, PI3K/AKT	NET formation	Inhibitor	(59)
	miR-26b-5p	MMP-8	NET formation	Inhibitor	(61)
	miR-5119	PD-L1	NET formation	Inhibitor	(62)
	miR-5106	Slc39a2	NET formation	Inhibitor	(63)

miRNA, microRNA; C/EBPα, CCAAT enhancer protein alpha; NFI-A, nuclear factor 1 A-type; SOCS3, suppressor of cytokine signaling 3; RUNX, runt-related transcription factor 1; GFI-1, growth factor independent-1; MEF2C, myocyte-specific enhancer factor 2C; IFN-γ, interferon γ; CBL, casitas B-lineage lymphoma; CDK2, cyclin-dependent kinase 2; ICAM-1, intercellular adhesion molecule-1; CtsB, Cathepsin B; NF-κB, nuclear factor-kappaB; MAPK, mitogen-activated protein kinase; NET, neutrophil extracellular trap; PAD4, peptidylarginine deiminase 4; PDK1, phosphoinositide-dependent protein kinase 1; AKT, protein kinase B; mTOR, mammalian target of rapamycin; JAGN1, junctional adhesion molecule-like protein; SIRT3, sirtuin 3; HMGB1, high-mobility group box 1; RAF-1, rapidly accelerated fibrosarcoma-1; PI3K, phosphatidylinositol 3-kinase; ERK, extracellular signal-regulated kinase; GPX3, glutathione peroxidase 3; MMP, matrix metalloproteinase; PD-L1, programmed death-ligand 1.

## 3 The miRNA-neutrophil axis in acute lung injury

Acute lung injury (ALI) and acute respiratory distress syndrome (ARDS) are prevalent among critically ill patients and represent acute inflammatory lung conditions with the existence of hypoxemia and bilateral lung infiltrates. There are many risk factors associated with ALI, including direct (pulmonary) injury (e.g., pneumonia, gastric aspiration, pulmonary contusion, alveolar hemorrhage, etc.) and indirect (extrapulmonary) injury (e.g., severe sepsis, transfusions, pancreatitis, and shock) (64). Although treatments have been updated, there are no specific therapeutic strategies for ALI and ARDS. Many ALI or ARDS patients need positive-pressure ventilation to maintain adequate oxygenation (65). Once lung tissue is damaged, the resident lung cells can attract neutrophils and macrophages into the airway microenvironment and trigger inflammatory responses (66). Neutrophils are activated to boost inflammation resolution and repair injured tissues in response to inflammatory stimuli. Excessive numbers of activated neutrophils lead to uncontrolled secretion of cytotoxic substances and eventually contribute to tissue injury (13). In ALI, excessive neutrophil recruitment and uncontrolled NET formation are central to tissue damage. Dysregulated NET formation exacerbates pulmonary damage via enhancing thromboinflammatory responses, disrupting endothelial integrity, and increasing vascular permeability. NETs are implicated in several forms of ALI, including those resulting from sepsis, coronavirus disease-19 (COVID-19), trauma, transfusion, ischemia-reperfusion, and mechanical ventilation (67). Novel therapeutic strategies that aim to block NET release or neutralize NET effects are being explored as potential interventions to mitigate NET-mediated lung injury (68).

### 3.1 The positive roles of miRNAs in neutrophil-linked acute lung injury

Macrophages are activated during tissue damage and undergo phenotypic and functional changes to participate in tissue repair and regeneration. Dysregulated macrophages contribute to the excessive release of inflammatory factors and destroy tissue regeneration. Macrophages can be activated into M1 macrophages that can release many pro-inflammatory molecules and M2 macrophages that can produce anti-inflammatory cytokines (69). miR-30d-5p expression is up-regulated in lung tissues obtained from mice undergoing cecal ligation and puncture (CLP), and its expression in exosomes derived from polymorphonuclear neutrophils (PMNs) is enhanced by TNF-α. TNF-α-treated PMNs deliver exosomal miR-30d-5p into macrophages and elevate miR-30d-5p expression in macrophages (70). Exosomal miR-30d-5p activates the NF-κB pathway via repressing SOCS1 and SIRT1, which can stimulate macrophage polarization into M1 types as well as strengthen macrophage pyroptosis. TNF-α-treated PMN-derived exosomal miR-30d-5p ultimately contributes to potentiating pulmonary inflammation and deteriorating sepsis-induced ALI. Suppressing miR-30d-5p mitigates lung injury caused by TNF-α-treated PMN exosomes or CLP through the inhibition of M1 macrophage activation and macrophage pyroptosis *in vivo* (70). miR-155 expression is increased by smoke exposure. Elevated miR-155 promotes lung neutrophil accumulation through repressing SOCS1, which can ultimately exaggerate lung injury induced by smoke inhalation (71). Transfusion-related acute lung injury (TRALI) is one of the severe transfusion consequences and occurs within 6 hours after transfusion. TRALI is characterized by sudden hypoxic respiratory failure and noncardiogenic pulmonary edema following blood product delivery (72). Increased NET formation is detected in plasma samples from TRALI patients (73). Previous studies indicated that activated neutrophils can produce excessive ROS and NETs, then impair pulmonary endothelium and contribute to pulmonary edema, resulting in deteriorating TRALI (74–76). Suppressing NET formation is a promising method to ameliorate TRALI (74, 77). miR-144 expression is up-regulated in peripheral blood samples collected from TRALI patients and TRALI mice compared to that in normal people and normal mice. Increased miR-144 activates NF-κB/C-X-C motif receptor 1 (CXCR1) pathway via repressing Krüppel‐like factor 2 (KLF2). Increased miR-144 finally enhances NET formation to exacerbate TRALI in mice (78). COVID-19 is a severe pulmonary disease caused by severe acute respiratory syndrome coronavirus 2 (SARS-CoV-2). Most COVID-19 patients undergo mild to moderate pneumonia-related lung injury, while some COVID-19 patients suffer from ARDS and need mechanical ventilation (79). Neutrophils protect lung tissues from infection in the early phase of COVID-19 but exhibit pathological roles in the later phase of viral infection, as evidenced by cytokine storm and uncontrolled immunothrombosis caused by excessive NET formation in infected lungs. Enhanced infiltration of dysfunctional neutrophils exacerbates alveolar epithelial damage and lung inflammation and eventually deteriorates COVID-19 (80, 81). Liao et al. used immunofluorescence staining to detect NET formation in response to the treatment of PMA and serum EVs from COVID-19 patients and found H3Cit co-localization with MPO in neutrophils. Subsequent research observed the elevated expression of miR-20b-5p in COVID-19 serum EVs and proved that pharmacological inhibition of miR-20b-5p significantly abrogates the capacity of COVID-19 EVs to induce NETs, as indicated by decreased H3Cit and MPO (82). Another study used a separate miRNA sequencing analysis to detect differentially expressed miRNAs of plasma EVs and found that miR-21 and miR-let-7b levels are significantly up-regulated, particularly in severe cases of COVID-19. miR-21 mimics and miR-let-7b mimics can be transferred into platelet EVs by electroporation. These EVs are effectively delivered into neutrophils to elevate miR-21 and miR-let-7b. Increased miR-21 and miR-let-7b activate nicotinamide-adenine dinucleotide phosphate (NADPH) oxidase 2 (NOX2) via promoting TLR7 and TLR8 expression. Eventually, platelet EV miR-21 and miR-let-7b stimulate SARS-CoV-2-triggered NET formation through increasing NADPH-associated ROS generation (83). Platelet EV miR-21 also enhances NF-κB activation through the same TLR pathways and results in potentiated release of IL-1β, TNF-α, and IL-8 in neutrophils. These increased inflammatory mediators further establish a pro-thrombotic feedback loop via platelet activation, which is correlated with the severity and death of COVID-19 (83).

### 3.2 The negative roles of miRNAs in neutrophil-linked acute lung injury

One study indicated that activated PMNs interact with epithelial cells under co-culture conditions and transfer microvesicles with miR-223 into epithelial cells to increase miR-223 expression in epithelial cells. Delivery of miR-223 from PMNs to epithelial cells can mitigate lung injury mediated by mechanical ventilation or infection via reducing Poly (adenosine diphosphate-ribose) polymerase-1 (PARP-1). PARP-1 deficiency or treatment of nanoparticles containing miR-223 mimics represses lung edema and pulmonary inflammation in mice (84). Feng et al. found that miR-223 upregulation dampens the differentiation of granulocyte precursors into Ly6G+ neutrophils as well as decreases the number of Ly6G+ neutrophils in the circulation. miR-223 knockout stimulates the activation of the NOD-like receptor protein 3 (NLRP3) inflammasome to increase IL-1β release, resulting in aggravating lung injury caused by mitochondrial damage-associated molecular patterns in mice (85). Consistent evidence shows that miR-223 deletion stimulates NET formation through enhancing neutrophil elastase, resulting in deteriorating ALI (86). miR-let-7b expression is significantly decreased in septic mice compared with normal mice. Overexpressing miR-let-7b suppresses the TLR4/NF-κB pathway to decline levels of pro-inflammatory cytokines (IL-6, IL-8, and TNF-α) and boost production of anti-inflammatory cytokine IL-10 in neutrophils. Up-regulated miR-let-7b ameliorates sepsis-induced pulmonary inflammation and increases survival rate via repressing neutrophil recruitment (87). miR-125a expression is lower in monocytes isolated from systemic lupus erythematosus patients than in monocytes from normal people. IL-16 is shown to facilitate CXCL10 expression of epithelial cells to promote neutrophil recruitment into the mouse lung and drive lung inflammation in response to pristane. Enhanced miR-125a restrains IL-16 expression to attenuate neutrophil accumulation in the mouse lung, which can hinder pristane-mediated alveolar hemorrhage *in vivo* (88). Another study reported that miR-125a knockout confers a protective effect against LPS-triggered lung damage via the suppression of granulopoiesis *in vivo*. Notably, this genetic intervention is also associated with a significant increase in survival rates in murine models of lethal septic shock (22). Mesenchymal stem cells (MSCs) represent one kind of somatic stem cells and possess the properties to self-renew and differentiate into multiple cell types. They can be isolated from several tissues, including bone marrow, adipose tissue, placenta, peripheral blood, and gingiva (89). Accumulative evidence indicates that MSCs and their exosomes are considered therapeutic tools to develop regenerative medicine to treat diseases, such as lung disorders, myocardial infarction, neurological disorders, and wound healing, etc. (90). miR-127-5p can be detected in exosomes isolated from bone marrow MSCs and can be delivered into mice. Exosomal miR-127-5p suppresses NET formation via reducing CD64, which can mitigate LPS-induced ALI *in vivo* (91). Primary graft dysfunction (PGD) acts as a severe kind of ALI following lung transplantation and remains the primary cause of early morbidity and mortality in response to lung transplantation. PGD occurs in approximately 15%-25% of patients within the first 72h post-transplantation and contributes to chronic lung allograft dysfunction (92, 93). NET formation is driven by platelet activation in PGD. Once released, NETs aggravate lung damage through direct cytotoxic effects, microvascular occlusion, and amplification of neutrophil-driven inflammation during PGD. Treatment with aspirin (a platelet inhibitor) or DNase I, which can repress NET formation, attenuates lung injury in mouse transplant models (94). miR-21 levels are decreased in bronchoalveolar lavage fluid (BALF) obtained from PGD patients compared with those in non-PGD patients. Overexpressing miR-21 ameliorates lung injury via repressing NET formation through targeting IL-12A in PGD rats undergoing lung transplantation (95).

**Figure 2:**
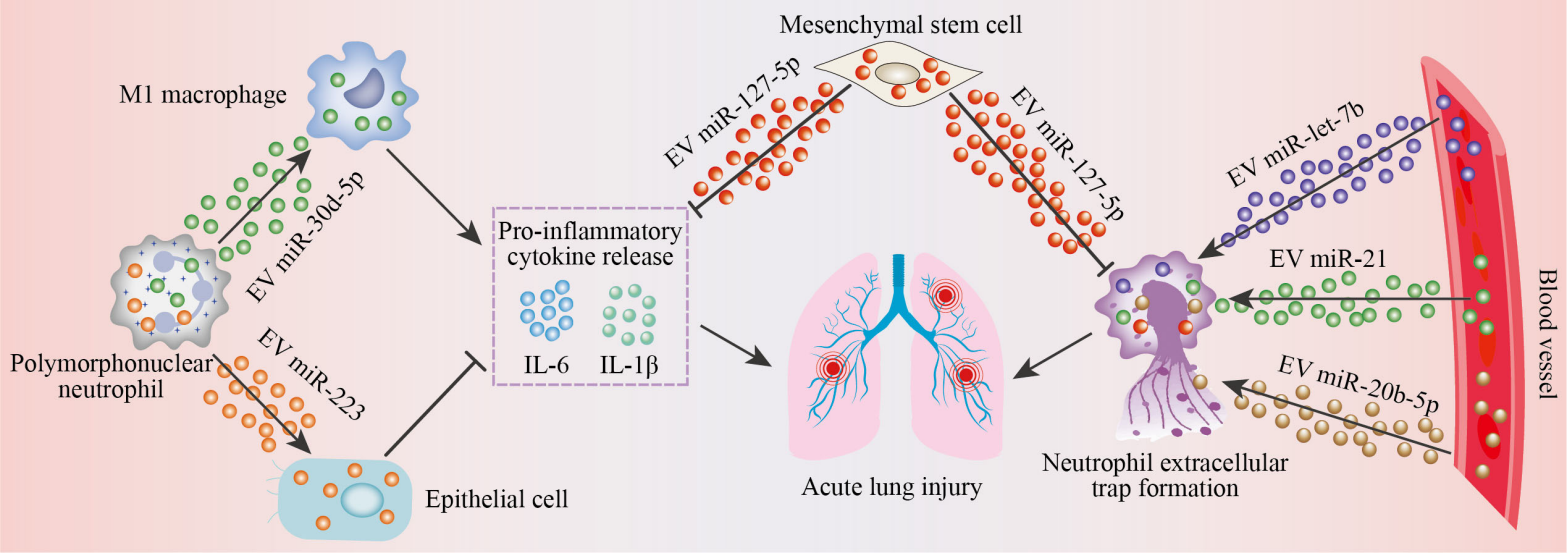
EV miRNAs regulate neutrophil-driven ALI. Excessive inflammatory cytokine (including IL-6 and IL-1β) release and NET formation are contributing factors to ALI development. Neutrophils deliver EV miR-30d-5p into M1 macrophages and transfer EV miR-223 into epithelial cells, which can facilitate and impede inflammation effector release, respectively. Circulating EV miR-20b-5p, miR-21, and miR-let-7b potentiate NET formation. EV miR-127-5p from MSCs can not only lessen inflammatory cytokine production but also abrogate NET formation.

Collectively, these studies described above demonstrate that miRNAs influence ALI pathogenesis through distinct neutrophil-related mechanisms. Some miRNAs exacerbate injury by promoting recruitment (miR-155 (71)) and NETs (miR-144 (78), miR-20b-5p (82), miR-21, and miR-let-7b (83)). Others amplify inflammation by polarizing macrophages toward a pro-inflammatory phenotype (miR-30d-5p (70)). Conversely, protective miRNAs attenuate ALI by suppressing NET formation (miR-223 (86), miR-127-5p (91), and miR-21 (95)), or reducing neutrophil recruitment (miR-let-7b (87) and miR-125a (88)). Cell-to-cell communication between neutrophils and target cells is governed by EV miRNAs. For example, neutrophils can deliver EV miRNAs into recipient cells (e.g., macrophages and epithelial cells) to mediate the production of inflammation effectors (**Figure 2**). The effects of EV miRNAs on the development of neutrophil-related ALI mainly depend on their source. For instance, EV miRNAs derived from MSCs and circulating EV miRNAs obtained from ALI patients exhibit inhibiting and promoting effects on NET formation, respectively (**Figure 2**).

## 4 The miRNA-neutrophil axis in chronic inflammatory lung diseases

### 4.1 Asthma

Asthma is a chronic inflammatory respiratory illness affecting an estimated 300 million people in the world and remains one of the causes of respiratory-related deaths (96). Asthma is characterized by reversible airway narrowing and airway hyperresponsiveness caused by nonspecific stimuli (e.g., air pollutants and allergens) (97). Eosinophilic asthma is correlated with type 2-high inflammation and is sensitive to the treatments of corticosteroids and bronchodilators. Neutrophilic asthma (2-low inflammation) is related to high neutrophilic infiltrations in airways and displays low sensitivity to inhaled corticosteroids (98). Neutrophils play a pivotal role in the pathogenesis of severe asthma through the release of extracellular DNA and NET formation. NETs contribute to airway inflammation by activating the inflammasome, leading to increased caspase-1 activity and elevated IL-1β levels in the airways. Asthma patients with elevated sputum extracellular DNA levels exhibit poorer asthma control, more frequent chronic mucus hypersecretion, and greater reliance on oral corticosteroids (99). Besides, elevated airway neutrophil levels are correlated with worse clinical outcomes, including accelerated lung function decline and increased hospitalizations (100). One study revealed that NET disruption significantly suppresses steroid resistance, suggesting that treatment with NET blockers may be a potential therapeutic strategy for neutrophil-associated asthma (101). Airway smooth muscle cells (ASMCs) are the main structural component in the airway and exhibit intrinsic abnormalities in asthma and undergo phenotype switching from a contractile to a synthetic-proliferative state. ASMCs serve as a passive effector of bronchoconstriction but as a central pathogenic driver integrating airway hyperresponsiveness, inflammation, and remodeling (102). One study displayed that the levels of miR-223-3p, miR-142-3p, and miR-629-3p are positively correlated with neutrophil percentages in the sputum of patients suffering from severe asthma. miR-629-3p mimics enhance miR-629-3p expression to elevate IL-8 levels in epithelial cells, which can attract circulating neutrophils into the lung in asthma (103). Serum exosomes loaded with miR-155 inhibitors decrease the number of BALF neutrophils and impede NETosis in asthmatic mice. Moreover, knockdown of miR-155 *in vivo* effectively abrogates neutrophilic airway inflammation (104). Proteomic analysis of neutrophil-derived exosomes has identified a diverse array of proteins implicated in immune modulation, intercellular adhesion, and signal transduction cascades. Neutrophils engage in sophisticated intercellular communication via the release of exosomes, and these exosomes serve as vehicles for the targeted delivery of pro−remodeling signals to ASMCs (105). One study indicated that neutrophil exosomes expressing downregulated miR-21 possess the potential to promote ASMC hyperproliferation, which is related to bronchial wall thickening in equine models of asthma (106). Serum exosomes loaded with miR-155 inhibitors decrease the number of BALF neutrophils and impede NETosis in asthmatic mice. Moreover, knockdown of miR-155 *in vivo* effectively abrogates neutrophilic airway inflammation (104). Further studies have shown that overexpression of miR-3164 inhibits PAD4 expression in neutrophils, thereby reducing neutrophil elastase and MPO to suppress NET formation. Under co-culture conditions, transfection of miR-3164 mimics in neutrophils reduces remodeling capacity, proliferative activity, and migratory ability of ASMCs treated with LPS and ATP, mediated through the suppression of TLR2/NF-κB signaling pathway activation (107). miR-4517 levels are decreased in the plasma of asthmatic individuals and are up-regulated within airway epithelial cells (AECs) following exposure to EVs derived from *Micrococcus luteus*. AECs carry EVs expressing miR-4517 into monocytes and block NLRP3 expression and IL-1β production, ultimately reducing neutrophilic airway inflammation via the inactivation of group 3 innate lymphoid cells (ILC3s) in asthma (108). Xu et al. suggested that enhanced miR-223 dampens lung infiltration of inflammatory cells (neutrophils, eosinophils, and lymphocytes) and pulmonary inflammation via suppressing NLRP3 inflammasome activity and IL-1β expression in neutrophilic asthma mice (109). Taken together, miRNAs regulate key pathological processes including neutrophil recruitment and NET formation in asthma. miR-629-3p and miR-155 show positive roles in asthma through positively influencing neutrophil infiltration (103) and NETs (104), respectively. While miRNAs miR-3164 (107), miR-4517 (108), and miR-223 (109) exhibit negative effects on asthma through inhibition of NETs or neutrophil recruitment.

### 4.2 Pulmonary fibrosis

Pulmonary fibrosis is a progressive and ultimately fatal interstitial lung disease characterized by the irreversible obliteration of alveolar structures and the consequent severe impairment of gas exchange (110). In preclinical research, murine models induced by bleomycin or silica instillation serve as the principal systems for investigating the molecular and cellular mechanisms driving pulmonary fibrosis pathogenesis (111, 112). A cornerstone event in pulmonary fibrogenesis is the transition of fibroblasts to myofibroblasts, which can drive the excessive synthesis and pathologic deposition of extracellular matrix components within the lung parenchyma, thereby leading to fibrotic tissue remodeling and progressive functional impairment (113, 114). Elevated neutrophil counts observed in both BALF and peripheral circulation are correlated with disease severity and worse prognosis in patients suffering from pulmonary fibrosis. On the one hand, neutrophil elastase promotes fibroblast proliferation and myofibroblast differentiation. On the other hand, NETs, composed of extracellular DNA and granular proteins, induce epithelial damage and enhance fibroblast activation (115). miR-7 expression is decreased in lung tissues isolated from mice with pulmonary fibrosis stimulated by NETs. Increased miR-7 inhibits Smad2 to abrogate the positive effects of TLR9 agonist on lung fibroblast proliferation and differentiation into myofibroblasts in response to the treatment of PMA-induced NETs *in vitro*. miR-7 overexpression is shown to attenuate the progression of NET-induced pulmonary fibrosis in mice (116). In summary, in pulmonary fibrosis, miRNAs can regulate fibroblast activation, which is strongly correlated with neutrophil-linked fibrosis remodeling.

### 4.3 Lung cancer

Lung cancer represents the most commonly diagnosed malignancy worldwide and remains the foremost cause of cancer mortality. Lung cancer is primarily classified into small cell lung cancer (SCLC), comprising approximately 15% of cases, and non−small cell lung cancer (NSCLC), which constitutes the majority at around 85%. NSCLC can be further subdivided into major histological subtypes, including lung adenocarcinoma, lung squamous cell carcinoma, and large cell carcinoma (117). The pathogenesis of lung cancer involves a complex interaction between genetic susceptibility and environmental factors, with well-established risk factors encompassing tobacco smoke, ionizing radiation, and exposure to various carcinogenic chemicals (118). Metastatic progression and disease recurrence represent the principal determinants of mortality in lung cancer patients. Notably, around two−thirds of patients present with detectable distant metastases at initial presentation, underscoring the pronounced therapeutic challenge in managing advanced−stage disease (119). Tumor-associated neutrophils (TANs), which infiltrate the tumor microenvironment, exhibit considerable functional plasticity and can be polarized into either tumor−promoting (N2 phenotype) or tumor−suppressing (N1 phenotype) states in response to local cytokine and chemokine signals (120–122). Consistent evidence from flow cytometry of cancer specimens from NSCLC patients demonstrates that TANs are the predominant immune cell population (123). When compared with circulating neutrophils in peripheral blood, TANs derived from human NSCLC tissues display a functional signature of activation, as evidenced by upregulated levels of intercellular adhesion molecule-1 (ICAM1/CD54) and chemokine receptors (CCR5, CCR7, CXCR3, and CXCR4), and reduced surface expression of L-selectin (CD62L) and the chemokine receptors CXCR1 and CXCR2 (124). TAN functional states are dynamically shaped by tumor-intrinsic cues, including hypoxia, histology (with higher TAN infiltration in squamous cell carcinoma than adenocarcinoma), and cancer cell-derived signals (125). TANs can accelerate lung tumor growth and enhance lung tumor resistance to radiotherapy (126). Understanding TAN functions is critical for developing precise therapeutic strategies that selectively target pro-tumorigenic TAN subpopulations while preserving or enhancing anti-tumor properties, rather than extensive and ineffective depletion of neutrophils (125, 127). Within the tumor microenvironment, a subset of TANs undergoes N2 polarization and expresses high levels of miR-941. Paracrine delivery of miR-941 to tumor cells impedes tumor cell apoptosis and promotes lung tumor cell invasion, migration, and proliferation. Up-regulated miR-941 silences tumor-suppressor forkhead box N4 (FOXN4) at mRNA levels to enhance TGF-β1 production, which creates a feed-forward loop that further amplifies N2 polarization of TANs (128). miR-320a expression is elevated in PMNs from heavy smokers with high risk for lung cancer development. PMNs deliver exogenous miR-320a into macrophages and raise intracellular miR-320a levels. Elevated miR-320a inhibits signal transducer and activator of transcription 4 (STAT4) to potentiate macrophage polarization toward an immunosuppressive M2 phenotype characterized by increased expression of M2 markers (e.g., IL-10, CD206, and CD163), thereby establishing an immunosuppressive microenvironment permissive for lung cancer initiation and progression (129). It is shown that neutrophil miR-574-3p and miR-26a-2-3p levels are related to pulmonary nodules and lung cancer stages in lung cancer patients, respectively. The combined use of neutrophil miR-574-3p and miR-26a-2-3p has high sensitivity and specificity to serve as circulating biomarkers to detect lung cancer (130). Altogether, in lung cancer, miRNAs mediate the pro-tumorigenic functions of TANs. miR-941 positively regulates N2 polarization and involves tumor cell aggressiveness (128), while PMN miR-320a fosters an immunosuppressive microenvironment by influencing M2 macrophage polarization (129).

## 5 Therapeutic opportunities and translational challenges

The growing understanding of how miRNAs govern neutrophil biology has naturally raised interest in their therapeutic potential. Modulating specific miRNAs to suppress neutrophil-driven inflammation or enhance host defense represents a promising strategy for treating inflammatory lung diseases. The following reviews current approaches, distinguishing between preclinical proof-of-concept studies and clinically actionable strategies, and discusses challenges (e.g., delivery, specificity, and safety) that should be overcome for clinical applications.

### 5.1 Preclinical proof-of-concept studies

The field of drug delivery has undergone significant evolution, driven by precise regulation of therapeutic agent distribution and release, minimizing systemic toxicity and enhancing therapeutic efficacy. This evolution builds upon a well-established foundation of synthetic delivery platforms, including liposomes, nanoparticles, dendrimers, microparticles, and nanocrystals, as extensively detailed in existing literature (131). Beyond these conventional systems, neutrophil-based delivery strategies have recently garnered considerable attention owing to the intrinsic biological properties of these immune cells, including innate capacity for rapid chemotaxis toward inflammatory sites, efficient phagocytic clearance, and responsive recruitment to sites of infection or tissue damage (132). Contemporary research has advanced neutrophil-mediated nano-delivery approaches through two principal modalities: the application of neutrophil membrane-coated nanoparticles and the engineering of whole neutrophils as living transport systems. These hybrid systems are designed to improve targeting accuracy, prolong circulatory half-life, and promote selective accumulation within pathological microenvironments (133, 134). It is shown that NOX4 can interact with dynamin-related protein 1 (Drp1) to modulate the activation of the NLRP3 inflammasome (135). Plasma miR-182-5p levels are down-regulated in ALI patients compared to normal people. NOX4 is increased in ALI patients and functions as one target of miR-182-5p. miR-182-5p can be loaded into neutrophil membrane-engineered *Panax ginseng* root-derived exosomes (N-exo). N-exo-miR-182-5p ameliorates LPS-induced lung injury and lung inflammation via suppressing NOX4/Drp1/NLRP3 pathway *in vivo* (136). Wang et al. reported that miR-125a-5p levels and miR-221-3p levels are elevated and decreased in serum EVs isolated from sepsis patients, respectively. Macrophage membrane nanoparticles can transfer miR-125a-5p inhibitor into macrophages to reduce miR-125a-5p and deliver miR-221-3p mimic into neutrophils to enhance miR-221-3p expression. Macrophage membrane nanoparticles containing miR-125a-5p inhibitor and miR-221-3p mimic mitigate lung injury and elevate survival rates in mice undergoing LPS-induced ALI (137). *Pseudomonas aeruginosa* is a prevalent opportunistic pathogen and is frequently responsible for hospital-acquired pneumonia, particularly ventilator-associated pneumonia. Acute pulmonary infection with this organism precipitates severe ARDS and ALI through mechanisms including epithelial and endothelial cytotoxicity, extensive alveolar neutrophil infiltration, and pronounced release of inflammatory mediators (138, 139). Tay et al. proved that the application of the antagonism of miR-328 can enhance the antimicrobial activity of macrophages and neutrophils to promote lung bacterial clearance in infected mice (140).

### 5.2 Clinically actionable strategies

Several approved drugs have been found to exert beneficial effects partly through miRNA modulation. Caffeine, a metabolic stimulant, is clinically employed in the management of bronchopulmonary dysplasia in very low-weight infants (141). Mechanistic studies reveal that caffeine declines miR-301b expression through the cyclic AMP (cAMP)/protein kinase A (PKA)/NF-κB pathway. The transcription factor c-Myb functions as a direct downstream target of miR-301b, and its genetic knockdown enhances bacterial burdens and deteriorates infection-related lung injury. Suppressing miR-301b ameliorates lung injury via enhancing neutrophil infiltration through c-Myb in mice suffering from the infection of *Pseudomonas aeruginosa* (142). Both budesonide (BUD) and N-acetylcysteine (NAC) have been shown to restrain LPS-related acute lung hyperinflation and inflammatory responses *in vivo* (143). One clinical study indicated that the combined use of BUD and acetylcysteine can effectively treat childhood pneumonia (144, 145). miR-196b-5p levels are increased by LPS treatment and decreased by the treatment of BUD and NAC. SOCS3 acts as a miR-196b-5p target, and its expression is enhanced by BUD and NAC. Combined administration of BUD and NAC represses neutrophil recruitment to alleviate LPS-induced ALI through the miR-196b-5p-SOCS3 axis *in vivo* (146). Capsaicin is a bioactive compound in red chili pepper and has been used clinically to alleviate pain correlated with knee osteoarthritis in patients (147) and reduce nasal reactivity and nasal congestion in patients with nonallergic rhinitis (148). Adel et al. proved that capsaicin downregulates miR-155-5p to diminish the expression of IL-1β, TNF-α, TGF-β1, PAD4, and neutrophil elastase, which can block NETosis production and alleviate lung damage in pulmonary fibrosis rats (149).

### 5.3 Translational challenges

Despite the abundance of preclinical evidence, translation of miRNA-based therapies into clinical practice for inflammatory lung diseases remains limited. Currently, no miRNA-targeted therapeutic has been approved for respiratory indications. The gap between preclinical experiments and clinical reality is attributable to several persistent challenges. The first concerns stability. Unmodified miRNAs are rapidly degraded by nucleases present in biological fluids. Although research reports suggest that chemical modification can enhance the stability of miRNA (150), there is insufficient clinical data to demonstrate its safety and efficacy. Second, effective delivery of miRNA mimics or inhibitors to specific cell types (e.g., neutrophils and alveolar cells) or specific organs (e.g., lung and liver) without off-target accumulation remains a major hurdle. While nanoparticle and engineered exosomes serve as promising drug delivery systems in animal models (151, 152), their immunogenicity and organ specificity in humans require rigorous evaluation. For instance, a clinical trial (NCT01829971) of MRX34 loading with miR-34a mimic was terminated because of serious immune side effects and lack of organ specificity (153). Third, off-target effects represent another fundamental concern. Given that each miRNA can potentially regulate multiple mRNAs, treatments of a single miRNA may produce unexpected consequences in non-target cell types or tissues. For example, miR-155 inhibition, while reducing NETosis (149), can also affect immune cell development or anti-tumor immunity (154). Therefore, safety or toxicity studies should be evaluated before clinical trials. Finally, reliable biomarkers are needed to identify patients most likely to respond to miRNA-based therapies and to monitor therapeutic response. Circulating EV miRNAs have been proposed (155), but standardized detection methods and cut-off values are yet to be established. In summary, while the miRNA-neutrophil axis offers compelling opportunities for therapeutic intervention in inflammatory lung diseases, realizing these potential demands requires continued innovation in delivery technology, rigorous evaluation of safety and specificity, and careful consideration of the complexities in translating preclinical findings to human patients. Future efforts should focus on developing targeted delivery vehicles, establishing patient stratification biomarkers based on miRNA signatures, and conducting rigorous safety and efficacy trials in relevant disease models.

## 6 Discussion and conclusions

Neutrophils are the quintessential first responders of the innate immune system, playing a dual role in host defense and tissue pathology. Their dysregulated activation, characterized by excessive infiltration, oxidative burst, and NET formation, constitutes a central pathogenic mechanism in inflammatory lung diseases such as ALI, severe asthma, and pulmonary fibrosis. TANs are different from circulating neutrophils and possess the capacity to polarize into tumor−promoting phenotypes or tumor−suppressing phenotypes in response to signals in the tumor microenvironment. In this review, we discuss how miRNAs can exert positive or negative roles in neutrophil biology (**Table 1**) and inflammatory lung diseases (**Table 2**). The miRNA-neutrophil axis encompasses an important regulatory role in both protective immunity and pathological inflammation in the lung. The studies reviewed here have displayed effects of miRNAs on granulopoiesis, recruitment, and NET formation, and have linked these mechanisms to ALI, asthma, pulmonary fibrosis, and lung cancer. In ALI, miRNAs predominantly regulate neutrophil recruitment and NET formation, which can contribute to tissue damage and pulmonary inflammation. In chronic diseases like asthma, pulmonary fibrosis, and lung cancer, miRNAs additionally modulate neutrophil infiltration, fibroblast activation, and immune polarization, linking neutrophil activity to airway remodeling and tumor progression. Cumulative evidence from the studies reviewed herein illustrates that miRNAs govern neutrophil biology across multiple levels, from granulopoiesis to effector functions, often through key transcription factors or signaling cascades. However, miRNAs often exert opposite effects in response to the cellular context, disease stage, distinct microenvironmental signals, or the specific downstream pathways. For instance, miR-223 promotes granulocytic differentiation via reducing NFI-A during steady-state hematopoiesis (16), but it can also show negative effects on granulopoiesis through MEF2C (28), suppresses neutrophil recruitment through inhibiting the NF-κB pathway (42), and represses NET formation by decreasing neutrophil elastase (56) under inflammatory conditions. miR-223 also attenuates ALI (85) and asthma (109) via abrogating NLRP3 inflammasome. miR-146a from keratinocytes (43) and alveolar cells (57) shows negative effects on neutrophil recruitment and NET formation. In contrast, miR-146a-5p from macrophage exosomes induces NET formation (54). Similarly, EV miR-30d-5p from PMNs promotes ALI through the NF-κB pathway (70), while PMN EV miR-223 represses ALI via targeting PARP-1 (84). Besides, elevated miR-21 blocks NET formation via targeting IL-12A (95). miR-let-7b up-regulation impedes neutrophilic inflammation and neutrophil recruitment via the interaction of the TLR4/NF-κB pathway (88). However, platelet EV miR-21 and miR-let-7b stimulate NET formation via TLR7 and TLR8 activation (83).

**Table 2:** Summary of miRNAs in neutrophil-linked lung diseases.

Disease	miRNA	Key target(s)/pathway	Outcome	Role	Reference
ALI	miR-30d-5p	SOCS1, SIRT1→NF-κB	M1 polarization, pyroptosis	Promoter	(70)
	miR-155	SOCS1	Neutrophil accumulation	Promoter	(71)
	miR-144	KLF2→NF-κB/CXCR1	NET formation	Promoter	(78)
	miR-20b-5p	H3Cit, MPO	NET formation	Promoter	(82)
	miR-21, miR-let-7b	TLR7, TLR8	NET formation	Promoter	(83)
	miR-223	PARP-1, NLRP3	Lung injury	Inhibitor	(84, 85)
		Neutrophil elastase	NET formation	Inhibitor	(86)
	miR-let-7b	TLR4/NF-κB	Neutrophil recruitment	Inhibitor	(87)
	miR-125a	IL-16	Neutrophil accumulation	Inhibitor	(88)
	miR-127-5p	CD64	NET formation	Inhibitor	(91)
	miR-21	IL-12A	NET formation	Inhibitor	(95)
	miR-182-5p	NOX4/Drp1/NLRP3	Lung inflammation	Inhibitor	(136)
	miR-301b	c-Myb	Neutrophil infiltration	Promoter	(142)
	miR-196b-5p	SOCS3	neutrophil recruitment	Promoter	(146)
Asthma	miR-3164	PAD4, TLR2/NF-κB	NET formation	Inhibitor	(107)
	miR-4517	NLRP3, IL-1β	ILC3 activation	Inhibitor	(108)
	miR-223	NLRP3, IL-1β	Neutrophil infiltration	Inhibitor	(109)
Pulmonary fibrosis	miR-7	Smad2	Fibroblast activation	Inhibitor	(116)
	miR-155-5p	IL-1β, TNF-α, TGF-β1, PAD4, neutrophil elastase	NETosis	Promoter	(149)
Lung cancer	miR-941	FOXN4→TGF-β1	N2 polarization	Promoter	(128)
	miR-320a	STAT4	M2 polarization	Promoter	(129)

miRNA, microRNA; ALI, acute lung injury; SOCS, suppressor of cytokine signaling; SIRT1, sirtuin 1; NF-κB, nuclear factor-kappaB; KLF2, Krüppel‐like factor 2; CXCR1, C-X-C motif receptor 1; NET, neutrophil extracellular trap; H3Cit, citrullinated histone 3; MPO, myeloperoxidase; TLR, toll-like receptor; PARP-1, Poly (adenosine diphosphate-ribose) polymerase-1; NLRP3, NOD-like receptor protein 3; NOX4, nicotinamide-adenine dinucleotide phosphate (NADPH) oxidase 4; Drp1, dynamin-related protein 1; PAD4, peptidylarginine deiminase 4; ILC3, group 3 innate lymphoid cell; TNF-α, tumor necrosis factor-α; TGF-β1, transforming growth factor beta 1; FOXN4, forkhead box N4; STAT4, signal transducer and activator of transcription 4.

Accumulating evidence synthesized in this review delineates a regulatory network centered on the miRNA-neutrophil axis, in which a select subset of hub miRNAs orchestrates neutrophil biology through intracellular signaling cascades. Notably, miR-223 emerges as a critical modulator of neutrophil functionality. miR-223 emerges as a common protective factor across ALI and asthma, primarily through its ability to suppress NLRP3 inflammasome activation (85, 109). Conversely, miR-155 acts as a pro-inflammatory node, enhancing NETs (50), promoting neutrophil recruitment (71), and inducing inflammatory cytokine secretion (149). At the pathway level, miRNAs converge upon conserved signaling cascades. For example, both miR-16-5p and miR-1696 modulate MAPK and PI3K/AKT pathways to influence NETs (58, 59). Both miR-223 (42) and miR-146a (43) repress the NF-κB pathway to impair neutrophil recruitment. Increased miR-let-7b impedes the NF-κB pathway to dampen neutrophil recruitment, showing protective effects on ALI (87). While miR-144 potentiates NETs via activating the NF-κB pathway, thus exacerbating TRALI (78). Accordingly, therapeutic strategies directed at these shared points, rather than individual miRNA species, may obtain enhanced efficacy in the management of inflammatory lung diseases.

Long noncoding RNAs (lncRNAs) represent another pivotal class of non-coding transcripts and function as competitive endogenous RNAs (ceRNAs) to impair miRNA-mediated repression on their targeted genes (156). In rats undergoing lung transplant ischemia-reperfusion, lncRNA metastasis-associated lung adenocarcinoma transcript 1 (MALAT1) expression is elevated. p300 overexpression promotes H3K27 acetylation enrichment on the promoter region of IL-8 and enhance IL-8expression. Suppressing MALAT1 downregulates p300-induced IL-8, resulting in alleviating ischemia-reperfusion-induced inflammatory injury via repressing neutrophil chemotaxis *in vivo* (157). LncRNA H19 is correlated with the development of PGD. Mechanistically, H19 augments KLF5 expression, which can increase CCL28 production of endothelial cells and subsequently stimulate neutrophil and macrophage recruitment. Genetic inhibition of H19 reduces pro-inflammatory effector secretion and dampens the infiltration of neutrophils and macrophages, ultimately alleviating lung injury in PGD mouse models (158). LncRNA colorectal neoplasia differentially expressed (CRNDE) expression is increased in exosomes isolated from LPS-treated neutrophils. Upon transfer to ASMCs, exosomal CRNDE activates the NF-κB pathway to accelerate ASMC migration and proliferation. *In vivo* Knockout of neutrophil CRNDE inhibits asthma progression in mice, as demonstrated by repressed hypertrophy and hyperplasia of the smooth muscle (159). LncRNA MIR503HG levels are decreased in lung cancer tissues compared to adjacent normal tissues. Restoration of MIR503HG represses NF-κB activation and then suppresses NLRP3 inflammasome, resulting in blocking NET-elicited lung cancer metastasis (160). Consistent with this, another study investigated that NETs abrogate MIR503HG to raise C/EBPβ expression, which can augment NLRP3 transcription to accelerate NSCLC metastasis (161). These studies indicated that the lncRNA-neutrophil axis is also involved in inflammatory lung diseases.

Despite significant progress in miRNAs and neutrophils, several critical questions remain unanswered. First, the majority of findings are derived from animal models or *in vitro* systems, and their direct relevance to human diseases remains to be established. For example, the zebrafish research identified miR-199-3p as a potent suppressor of neutrophil chemotaxis via CDK2 (37), whether this axis operates in human neutrophils during inflammatory lung diseases remains to be determined. Second, miRNA expression and neutrophil function change dynamically during disease initiation, progression, or resolution. However, most studies assess miRNA levels and neutrophil effects at a single time point. Besides, most animal studies employ acute injury models such as LPS administration (91) or CLP (70), which replicate certain features of human ALI but fail to capture the heterogeneity, chronicity, and comorbid conditions characteristic of clinical populations. Third, many studies rely on up-regulation or knockdown approaches that may not reflect physiological miRNA levels, potentially exaggerating or covering original regulatory effects. There are also several gaps in research associated with miRNAs and neutrophils. First, most studies have analyzed single neutrophil populations or mixed cell types. Given the growing understanding of neutrophil heterogeneity (e.g., N1, N2, aged, and fresh neutrophils) (162), single-cell resolution studies are needed to map miRNA expression across neutrophil subsets and link specific miRNAs to distinct functional states. Second, the downstream targets or signaling pathways of miRNAs in neutrophils are increasingly characterized (163). The signals that control miRNA expression in neutrophils remain poorly understood. Identifying the transcription factors, epigenetic regulators, and microenvironmental signals that govern miRNA expression would be essential for therapeutic targeting. Third, miRNAs are embedded in complex regulatory networks involving transcription factors, lncRNAs, and other non-coding RNAs (164). Integrative approaches combining multi-omics data and computational modeling would be necessary to capture this complexity.

## References

[1] Qi Y Yang B Ouyang H Wang X Li C Li L . Advanced Nanotherapies for Precision Treatment of Inflammatory Lung Diseases. Bioactive materials. (2025) 53:329–65. doi: 10.1016/j.bioactmat.2025.07.028, PMID: 40727485 PMC12302282

[2] Hussain MS Goyal A Goyal K RJ S Nellore J Shahwan M . Targeting Cxcr2 Signaling in Inflammatory Lung Diseases: Neutrophil-Driven Inflammation and Emerging Therapies. Naunyn-Schmiedeberg’s Arch Pharmacol. (2025) 398:9583–607. doi: 10.1007/s00210-025-03970-x, PMID: 40047857

[3] Zhang F Xia Y Su J Quan F Zhou H Li Q . Neutrophil Diversity and Function in Health and Disease. Signal transduction targeted Ther. (2024) 9:343. doi: 10.1038/s41392-024-02049-y, PMID: 39638788 PMC11627463

[4] Herro R Grimes HL . The Diverse Roles of Neutrophils from Protection to Pathogenesis. Nat Immunol. (2024) 25:2209–19. doi: 10.1038/s41590-024-02006-5, PMID: 39567761

[5] Aroca-Crevillén A Vicanolo T Ovadia S Hidalgo A . Neutrophils in Physiology and Pathology. Annu Rev Pathol. (2024) 19:227–59. doi: 10.1146/annurev-pathmechdis-051222-015009, PMID: 38265879 PMC11060889

[6] Kim H Lee YY Kim VN . The Biogenesis and Regulation of Animal Micrornas. Nat Rev Mol Cell Biol. (2025) 26:276–96. doi: 10.1038/s41580-024-00805-0, PMID: 39702526

[7] Maji RK Leisegang MS Boon RA Schulz MH . Revealing Microrna Regulation in Single Cells. Trends genetics: TIG. (2025) 41:522–36. doi: 10.1016/j.tig.2024.12.009, PMID: 39863489

[8] Vladimirov S Tomasevic M Popov N Munjas J de Gonzalo-Calvo D Sopic M . The Converging Roles of Micrornas and Lipid Metabolism in Atherosclerotic Cardiovascular Disease and Cancer. Semin Cancer Biol. (2025) 114:41–59. doi: 10.1016/j.semcancer.2025.06.005, PMID: 40494407

[9] Wang P Lai D Jin L Xue Y . Roles of Micrornas in Acute Lung Injury and Acute Respiratory Distress Syndrome: Mechanisms and Clinical Potential. Front Immunol. (2025) 16:1570128. doi: 10.3389/fimmu.2025.1570128, PMID: 41080597 PMC12507827

[10] Lim SY Boyd SC Diefenbach RJ Rizos H . Circulating Micrornas: Functional Biomarkers for Melanoma Prognosis and Treatment. Mol Cancer. (2025) 24:99. doi: 10.1186/s12943-025-02298-7, PMID: 40156012 PMC11951542

[11] Weidner J Bartel S Kılıç A Zissler UM Renz H Schwarze J . Spotlight on Micrornas in Allergy and Asthma. Allergy. (2021) 76:1661–78. doi: 10.1111/all.14646, PMID: 33128813 PMC8246745

[12] Wei Y Gong W Wei Y Jiang X Li C Ye R . Decoding the Lncrna-Mirna-Mrna Network in Sepsis-Induced Lung Injury: From Pathogenesis to Extracellular Vesicle-Based Therapy. Front Immunol. (2026) 17:1701440. doi: 10.3389/fimmu.2026.1701440, PMID: 41685305 PMC12890660

[13] Ye S Ma L Chi Y Liu N Liu Y Wei W . Targeting Neutrophil Dysfunction in Acute Lung Injury: Insights from Active Components of Chinese Medicine. Phytomedicine: Int J phytotherapy phytopharmacol. (2025) 141:156664. doi: 10.1016/j.phymed.2025.156664, PMID: 40121883

[14] Lin WC Fessler MB . Regulatory Mechanisms of Neutrophil Migration from the Circulation to the Airspace. Cell Mol Life sciences: CMLS. (2021) 78:4095–124. doi: 10.1007/s00018-021-03768-z, PMID: 33544156 PMC7863617

[15] Kalafati L Hatzioannou A Hajishengallis G Chavakis T . The Role of Neutrophils in Trained Immunity. Immunol Rev. (2023) 314:142–57. doi: 10.1111/imr.13142, PMID: 36190144 PMC10050090

[16] Fazi F Rosa A Fatica A Gelmetti V De Marchis ML Nervi C . A Minicircuitry Comprised of Microrna-223 and Transcription Factors Nfi-a and C/Ebpalpha Regulates Human Granulopoiesis. Cell. (2005) 123:819–31. doi: 10.1016/j.cell.2005.09.023, PMID: 16325577

[17] Vian L Di Carlo M Pelosi E Fazi F Santoro S Cerio AM . Transcriptional Fine-Tuning of Microrna-223 Levels Directs Lineage Choice of Human Hematopoietic Progenitors. Cell Death differentiation. (2014) 21:290–301. doi: 10.1038/cdd.2013.145, PMID: 24141720 PMC3890951

[18] Pulikkan JA Dengler V Peramangalam PS Peer Zada AA Müller-Tidow C Bohlander SK . Cell-Cycle Regulator E2f1 and Microrna-223 Comprise an Autoregulatory Negative Feedback Loop in Acute Myeloid Leukemia. Blood. (2010) 115:1768–78. doi: 10.1182/blood-2009-08-240101, PMID: 20029046 PMC2832809

[19] Pulikkan JA Peramangalam PS Dengler V Ho PA Preudhomme C Meshinchi S . C/Ebpα Regulated Microrna-34a Targets E2f3 During Granulopoiesis and Is Down-Regulated in Aml with Cebpa Mutations. Blood. (2010) 116:5638–49. doi: 10.1182/blood-2010-04-281600, PMID: 20889924 PMC3031410

[20] Katzerke C Madan V Gerloff D Bräuer-Hartmann D Hartmann JU Wurm AA . Transcription Factor C/Ebpα-Induced Microrna-30c Inactivates Notch1 During Granulopoiesis and Is Downregulated in Acute Myeloid Leukemia. Blood. (2013) 122:2433–42. doi: 10.1182/blood-2012-12-472183, PMID: 23974200 PMC3790511

[21] Sun YM Lin KY Chen YQ . Diverse Functions of Mir-125 Family in Different Cell Contexts. J Hematol Oncol. (2013) 6:6. doi: 10.1186/1756-8722-6-6, PMID: 23321005 PMC3566921

[22] Qin Y Wu L Ouyang Y Zhou P Zhou H Wang Y . Mir-125a Is a Critical Modulator for Neutrophil Development. PloS Genet. (2017) 13:e1007027. doi: 10.1371/journal.pgen.1007027, PMID: 28976973 PMC5643141

[23] Zhao H Wang X Yi P Si Y Tan P He J . Ksrp Specifies Monocytic and Granulocytic Differentiation through Regulating Mir-129 Biogenesis and Runx1 Expression. Nat Commun. (2017) 8:1428. doi: 10.1038/s41467-017-01425-3, PMID: 29127290 PMC5681548

[24] Häger M Pedersen CC Larsen MT Andersen MK Hother C Grønbæk K . Microrna-130a-Mediated Down-Regulation of Smad4 Contributes to Reduced Sensitivity to Tgf-β1 Stimulation in Granulocytic Precursors. Blood. (2011) 118:6649–59. doi: 10.1182/blood-2011-03-339978, PMID: 22028478

[25] Larsen MT Häger M Glenthøj A Asmar F Clemmensen SN Mora-Jensen H . Mirna-130a Regulates C/Ebp-ϵ Expression During Granulopoiesis. Blood. (2014) 123:1079–89. doi: 10.1182/blood-2013-08-523233, PMID: 24398327

[26] Wurm AA Zjablovskaja P Kardosova M Gerloff D Bräuer-Hartmann D Katzerke C . Disruption of the C/Ebpα-Mir-182 Balance Impairs Granulocytic Differentiation. Nat Commun. (2017) 8:46. doi: 10.1038/s41467-017-00032-6, PMID: 28663557 PMC5491528

[27] Velu CS Baktula AM Grimes HL . Gfi1 Regulates Mir-21 and Mir-196b to Control Myelopoiesis. Blood. (2009) 113:4720–8. doi: 10.1182/blood-2008-11-190215, PMID: 19278956 PMC2680372

[28] Johnnidis JB Harris MH Wheeler RT Stehling-Sun S Lam MH Kirak O . Regulation of Progenitor Cell Proliferation and Granulocyte Function by Microrna-223. Nature. (2008) 451:1125–9. doi: 10.1038/nature06607, PMID: 18278031

[29] Kolaczkowska E Kubes P . Neutrophil Recruitment and Function in Health and Inflammation. Nat Rev Immunol. (2013) 13:159–75. doi: 10.1038/nri3399, PMID: 23435331

[30] Zhang N Tang W Torres L Wang X Ajaj Y Zhu L . Cell Surface Rnas Control Neutrophil Recruitment. Cell. (2024) 187:846–60.e17. doi: 10.1016/j.cell.2023.12.033, PMID: 38262409 PMC10922858

[31] Fan HB Liu YJ Wang L Du TT Dong M Gao L . Mir-142-3p Acts as an Essential Modulator of Neutrophil Development in Zebrafish. Blood. (2014) 124:1320–30. doi: 10.1182/blood-2013-12-545012, PMID: 24990885

[32] Kuang L Wu L Li Y . Extracellular Vesicles in Tumor Immunity: Mechanisms and Novel Insights. Mol Cancer. (2025) 24:45. doi: 10.1186/s12943-025-02233-w, PMID: 39953480 PMC11829561

[33] Li J Wang J Chen Z . Emerging Role of Exosomes in Cancer Therapy: Progress and Challenges. Mol Cancer. (2025) 24:13. doi: 10.1186/s12943-024-02215-4, PMID: 39806451 PMC11727182

[34] Akbar N Braithwaite AT Corr EM Koelwyn GJ van Solingen C Cochain C . Rapid Neutrophil Mobilization by Vcam-1+ Endothelial Cell-Derived Extracellular Vesicles. Cardiovasc Res. (2023) 119:236–51. doi: 10.1093/cvr/cvac012, PMID: 35134856 PMC10022859

[35] Wu X Liu H Hu Q Wang J Zhang S Cui W . Astrocyte-Derived Extracellular Vesicular Mir-143-3p Dampens Autophagic Degradation of Endothelial Adhesion Molecules and Promotes Neutrophil Transendothelial Migration after Acute Brain Injury. Advanced Sci (Weinheim Baden-Wurttemberg Germany). (2024) 11:e2305339. doi: 10.1002/advs.202305339, PMID: 38044319 PMC10837358

[36] Zhu Y Liu L Chu L Lan J Wei J Li W . Microscopic Polyangiitis Plasma-Derived Exosomal Mir-1287-5p Induces Endothelial Inflammatory Injury and Neutrophil Adhesion by Targeting Cbl. PeerJ. (2023) 11:e14579. doi: 10.7717/peerj.14579, PMID: 36726727 PMC9885867

[37] Hsu AY Wang D Liu S Lu J Syahirah R Bennin DA . Phenotypical Microrna Screen Reveals a Noncanonical Role of Cdk2 in Regulating Neutrophil Migration. Proc Natl Acad Sci United States America. (2019) 116:18561–70. doi: 10.1073/pnas.1905221116, PMID: 31451657 PMC6744913

[38] Hsu AY Wang D Gurol T Zhou W Zhu X Lu HY . Overexpression of Microrna-722 Fine-Tunes Neutrophilic Inflammation by Inhibiting Rac2 in Zebrafish. Dis Models Mech. (2017) 10:1323–32. doi: 10.1242/dmm.030791, PMID: 28954734 PMC5719257

[39] Hsu AY Liu S Syahirah R Brasseale KA Wan J Deng Q . Inducible Overexpression of Zebrafish Microrna-722 Suppresses Chemotaxis of Human Neutrophil Like Cells. Mol Immunol. (2019) 112:206–14. doi: 10.1016/j.molimm.2019.06.001, PMID: 31176200 PMC6659728

[40] Liu Y Feng Y Kong X Wei Y Zhan M Wang J . A Microrna Sponge, Linc02193, Promotes Neutrophil Activation by Upregulating Icam1 and Is Correlated with Anca-Associated Vasculitis. Rheumatol (Oxford England). (2024) 63:2295–306. doi: 10.1093/rheumatology/kead605, PMID: 37963065

[41] Wang D Wang T Kim D Tan S Liu S Wan J . Microrna-375 Modulates Neutrophil Chemotaxis Via Targeting Cathepsin B in Zebrafish. Fish shellfish Immunol. (2024) 154:109933. doi: 10.1016/j.fsi.2024.109933, PMID: 39343064 PMC11561466

[42] Zhou W Pal AS Hsu AY Gurol T Zhu X Wirbisky-Hershberger SE . Microrna-223 Suppresses the Canonical Nf-κb Pathway in Basal Keratinocytes to Dampen Neutrophilic Inflammation. Cell Rep. (2018) 22:1810–23. doi: 10.1016/j.celrep.2018.01.058, PMID: 29444433 PMC5839657

[43] Meisgen F Xu Landén N Wang A Réthi B Bouez C Zuccolo M . Mir-146a Negatively Regulates Tlr2-Induced Inflammatory Responses in Keratinocytes. J Invest Dermatol. (2014) 134:1931–40. doi: 10.1038/jid.2014.89, PMID: 24670381

[44] Kivihall A Aab A Soja J Sładek K Sanak M Altraja A . Reduced Expression of Mir-146a in Human Bronchial Epithelial Cells Alters Neutrophil Migration. Clin Trans Allergy. (2019) 9:62. doi: 10.1186/s13601-019-0301-8, PMID: 31798831 PMC6880603

[45] Murata K Yoshitomi H Furu M Ishikawa M Shibuya H Ito H . Microrna-451 Down-Regulates Neutrophil Chemotaxis Via P38 Mapk. Arthritis Rheumatol (Hoboken NJ). (2014) 66:549–59. doi: 10.1002/art.38269, PMID: 24574214

[46] Yao M Fang C Wang Z Guo T Wu D Ma J . Mir-328-3p Targets Tlr2 to Ameliorate Oxygen-Glucose Deprivation Injury and Neutrophil Extracellular Trap Formation in Huvecs Via Inhibition of the Nf-κb Signaling Pathway. PloS One. (2024) 19:e0299382. doi: 10.1371/journal.pone.0299382, PMID: 38394259 PMC10889604

[47] Gao F Peng H Gou R Zhou Y Ren S Li F . Exploring Neutrophil Extracellular Traps: Mechanisms of Immune Regulation and Future Therapeutic Potential. Exp Hematol Oncol. (2025) 14:80. doi: 10.1186/s40164-025-00670-3, PMID: 40442839 PMC12123823

[48] Zhang J Miao C Zhang H . Targeting Neutrophil Extracellular Traps in Cancer Progression and Metastasis. Theranostics. (2025) 15:5846–69. doi: 10.7150/thno.111096, PMID: 40365275 PMC12068306

[49] Wang H Kim SJ Lei Y Wang S Wang H Huang H . Neutrophil Extracellular Traps in Homeostasis and Disease. Signal transduction targeted Ther. (2024) 9:235. doi: 10.1038/s41392-024-01933-x, PMID: 39300084 PMC11415080

[50] Hawez A Al-Haidari A Madhi R Rahman M Thorlacius H . Mir-155 Regulates Pad4-Dependent Formation of Neutrophil Extracellular Traps. Front Immunol. (2019) 10:2462. doi: 10.3389/fimmu.2019.02462, PMID: 31736940 PMC6838784

[51] Hawez A Taha D Algaber A Madhi R Rahman M Thorlacius H . Mir-155 Regulates Neutrophil Extracellular Trap Formation and Lung Injury in Abdominal Sepsis. J leukocyte Biol. (2022) 111:391–400. doi: 10.1002/jlb.3a1220-789rr, PMID: 34114683

[52] Jiao Y Li W Wang W Tong X Xia R Fan J . Platelet-Derived Exosomes Promote Neutrophil Extracellular Trap Formation During Septic Shock. Crit Care (London England). (2020) 24:380. doi: 10.1186/s13054-020-03082-3, PMID: 32600436 PMC7322900

[53] Wang L Zhu Z Liao Y Zhang L Yu Z Yang R . Host Liver-Derived Extracellular Vesicles Deliver Mir-142a-3p Induces Neutrophil Extracellular Traps Via Targeting Wasl to Block the Development of Schistosoma Japonicum. Mol therapy: J Am Soc Gene Ther. (2022) 30:2092–107. doi: 10.1016/j.ymthe.2022.03.016, PMID: 35351657 PMC9092393

[54] Liu Z Zhang W Li Y Fu Y Zhang Y . Macrophage-Derived Exosome Mir-146a-5p Modulates Pnkp/Ddost/Jagn1 Complex to Regulate Nets Formation in Atherosclerosis. Cell Signaling. (2025) 136:112176. doi: 10.1016/j.cellsig.2025.112176, PMID: 41109654

[55] Chen L Hu L Li Q Ma J Li H . Exosome-Encapsulated Mir-505 from Ox-Ldl-Treated Vascular Endothelial Cells Aggravates Atherosclerosis by Inducing Net Formation. Acta Biochim Biophys Sin. (2019) 51:1233–41. doi: 10.1093/abbs/gmz123, PMID: 31768526

[56] Ye D Yao J Du W Chen C Yang Y Yan K . Neutrophil Extracellular Traps Mediate Acute Liver Failure in Regulation of Mir-223/Neutrophil Elastase Signaling in Mice. Cell Mol Gastroenterol Hepatol. (2022) 14:587–607. doi: 10.1016/j.jcmgh.2022.05.012, PMID: 35660025 PMC9307949

[57] Hsieh YT Chou YC Kuo PY Tsai HW Yen YT Shiau AL . Down-Regulated Mir-146a Expression with Increased Neutrophil Extracellular Traps and Apoptosis Formation in Autoimmune-Mediated Diffuse Alveolar Hemorrhage. J Biomed Sci. (2022) 29:62. doi: 10.1186/s12929-022-00849-4, PMID: 36028828 PMC9413930

[58] Yin K Cui Y Qu Y Zhang J Zhang H Lin H . Hydrogen Sulfide Upregulates Mir-16-5p Targeting Pik3r1 and Raf1 to Inhibit Neutrophil Extracellular Trap Formation in Chickens. Ecotoxicol Environ Saf. (2020) 194:110412. doi: 10.1016/j.ecoenv.2020.110412, PMID: 32155482

[59] Yang Z Wang S Yin K Zhang Q Li S . Mir-1696/Gpx3 Axis Is Involved in Oxidative Stress Mediated Neutrophil Extracellular Traps Inhibition in Chicken Neutrophils. J Cell Physiol. (2021) 236:3688–99. doi: 10.1002/jcp.30105, PMID: 33044016

[60] Yang B Huang X Xu S Li L Wu W Dai Y . Decreased Mir-4512 Levels in Monocytes and Macrophages of Individuals with Systemic Lupus Erythematosus Contribute to Innate Immune Activation and Neutrsophil Netosis by Targeting Tlr4 and Cxcl2. Front Immunol. (2021) 12:756825. doi: 10.3389/fimmu.2021.756825, PMID: 34721432 PMC8552026

[61] Rui S Dai L Zhang X He M Xu F Wu W . Exosomal Mirna-26b-5p from Prp Suppresses Nets by Targeting Mmp-8 to Promote Diabetic Wound Healing. J Controlled release: Off J Controlled Release Soc. (2024) 372:221–33. doi: 10.1016/j.jconrel.2024.06.050, PMID: 38909697

[62] Yang Y Yang L Yang Y Deng H Su S Xia Y . Bacteroides Fragilis-Derived Outer Membrane Vesicles Deliver Mir-5119 and Alleviate Colitis by Targeting Pd-L1 to Inhibit Gsdmd-Mediated Neutrophil Extracellular Trap Formation. Advanced Sci (Weinheim Baden-Wurttemberg Germany). (2025) 12:e00781. doi: 10.1002/advs.202500781, PMID: 40558568 PMC12462929

[63] Yang Y Yang L Deng H Liu Y Wu J Yang Y . Coptis Chinensis-Derived Extracellular Vesicle-Like Nanoparticles Delivered Mirna-5106 Suppresses Nets by Restoring Zinc Homeostasis to Alleviate Colitis. J nanobiotechnol. (2025) 23:444. doi: 10.1186/s12951-025-03466-z, PMID: 40517238 PMC12166623

[64] Lai X Zhong J Zhang B Zhu T Liao R . Exosomal Non-Coding Rnas: Novel Regulators of Macrophage-Linked Intercellular Communication in Lung Cancer and Inflammatory Lung Diseases. Biomolecules. (2023) 13. doi: 10.3390/biom13030536, PMID: 36979471 PMC10046066

[65] Zheng Y Zhao Y Zhang S Wang M Wang X Chen XL . Advances in Natural Product-Based Nanoparticles for the Treatment of Acute Lung Injury. Materials Today Bio. (2025) 35:102486. doi: 10.1016/j.mtbio.2025.102486, PMID: 41255417 PMC12621474

[66] Ren Z Zheng Z Feng X . Role of Gut Microbes in Acute Lung Injury/Acute Respiratory Distress Syndrome. Gut Microbes. (2024) 16:2440125. doi: 10.1080/19490976.2024.2440125, PMID: 39658851 PMC11639474

[67] Scozzi D Liao F Krupnick AS Kreisel D Gelman AE . The Role of Neutrophil Extracellular Traps in Acute Lung Injury. Front Immunol. (2022) 13:953195. doi: 10.3389/fimmu.2022.953195, PMID: 35967320 PMC9374003

[68] Du Y Chen Y Li F Mao Z Ding Y Wang W . Genetically Engineered Cellular Nanovesicle as Targeted Dnase I Delivery System for the Clearance of Neutrophil Extracellular Traps in Acute Lung Injury. Advanced Sci (Weinheim Baden-Wurttemberg Germany). (2023) 10:e2303053. doi: 10.1002/advs.202303053, PMID: 37759381 PMC10646266

[69] Guan F Wang R Yi Z Luo P Liu W Xie Y . Tissue Macrophages: Origin, Heterogenity, Biological Functions, Diseases and Therapeutic Targets. Signal transduction targeted Ther. (2025) 10:93. doi: 10.1038/s41392-025-02124-y, PMID: 40055311 PMC11889221

[70] Jiao Y Zhang T Zhang C Ji H Tong X Xia R . Exosomal Mir-30d-5p of Neutrophils Induces M1 Macrophage Polarization and Primes Macrophage Pyroptosis in Sepsis-Related Acute Lung Injury. Crit Care (London England). (2021) 25:356. doi: 10.1186/s13054-021-03775-3, PMID: 34641966 PMC8507252

[71] Zhang Y Xie Y Zhang L Zhao H . Microrna-155 Participates in Smoke-Inhalation-Induced Acute Lung Injury through Inhibition of Socs-1. Molecules (Basel Switzerland). (2020) 25. doi: 10.3390/molecules25051022, PMID: 32106541 PMC7179228

[72] Rayatdoost F Kapur R . Transfusion-Related Acute Lung Injury: Experimental Models to Study Pathogenesis and Therapeutic Strategies. Curr Opin Immunol. (2025) 96:102650. doi: 10.1016/j.coi.2025.102650, PMID: 40865410

[73] van der Velden S van Osch TLJ Seghier A Bentlage AEH Mok JY Geerdes DM . Complement Activation Drives Antibody-Mediated Transfusion-Related Acute Lung Injury Via Macrophage Trafficking and Formation of Nets. Blood. (2024) 143:79–91. doi: 10.1182/blood.2023020484, PMID: 37801721

[74] Liu Y Wang R Song C Ding S Zuo Y Yi K . Crosstalk between Neutrophil Extracellular Traps and Immune Regulation: Insights into Pathobiology and Therapeutic Implications of Transfusion-Related Acute Lung Injury. Front Immunol. (2023) 14:1324021. doi: 10.3389/fimmu.2023.1324021, PMID: 38162674 PMC10755469

[75] Fang X Mo C Zheng L Gao F Xue FS Zheng X . Transfusion-Related Acute Lung Injury: From Mechanistic Insights to Therapeutic Strategies. Advanced Sci (Weinheim Baden-Wurttemberg Germany). (2025) 12:e2413364. doi: 10.1002/advs.202413364, PMID: 39836498 PMC11923913

[76] Yu Y Lian Z . Update on Transfusion-Related Acute Lung Injury: An Overview of Its Pathogenesis and Management. Front Immunol. (2023) 14:1175387. doi: 10.3389/fimmu.2023.1175387, PMID: 37251400 PMC10213666

[77] Caudrillier A Kessenbrock K Gilliss BM Nguyen JX Marques MB Monestier M . Platelets Induce Neutrophil Extracellular Traps in Transfusion-Related Acute Lung Injury. J Clin Invest. (2012) 122:2661–71. doi: 10.1172/jci61303, PMID: 22684106 PMC3386815

[78] Le A Wu Y Liu W Wu C Hu P Zou J . Mir-144-Induced Klf2 Inhibition and Nf-Kappab/Cxcr1 Activation Promote Neutrophil Extracellular Trap-Induced Transfusion-Related Acute Lung Injury. J Cell Mol Med. (2021) 25:6511–23. doi: 10.1111/jcmm.16650, PMID: 34120407 PMC8278117

[79] Lamers MM Haagmans BL . Sars-Cov-2 Pathogenesis. Nat Rev Microbiol. (2022) 20:270–84. doi: 10.1038/s41579-022-00713-0, PMID: 35354968

[80] Middleton EA He XY Denorme F Campbell RA Ng D Salvatore SP . Neutrophil Extracellular Traps Contribute to Immunothrombosis in Covid-19 Acute Respiratory Distress Syndrome. Blood. (2020) 136:1169–79. doi: 10.1182/blood.2020007008, PMID: 32597954 PMC7472714

[81] Barnes BJ Adrover JM Baxter-Stoltzfus A Borczuk A Cools-Lartigue J Crawford JM . Targeting Potential Drivers of Covid-19: Neutrophil Extracellular Traps. J Exp Med. (2020) 217. doi: 10.1084/jem.20200652, PMID: 32302401 PMC7161085

[82] Liao Y Liu Y Li D Luo S Huang Y Wu J . Covid-19 Patient Serum-Derived Extracellular Vesicles Deliver Mir-20b-5p Induces Neutrophil Extracellular Traps. Cell communication signaling: CCS. (2025) 23:93. doi: 10.1186/s12964-025-02095-1, PMID: 39962581 PMC11834185

[83] Liao TL Liu HJ Chen DY Tang KT Chen YM Liu PY . Sars-Cov-2 Primed Platelets-Derived Micrornas Enhance Nets Formation by Extracellular Vesicle Transmission and Tlr7/8 Activation. Cell communication signaling: CCS. (2023) 21:304. doi: 10.1186/s12964-023-01345-4, PMID: 37904132 PMC10614402

[84] Neudecker V Brodsky KS Clambey ET Schmidt EP Packard TA Davenport B . Neutrophil Transfer of Mir-223 to Lung Epithelial Cells Dampens Acute Lung Injury in Mice. Sci Trans Med. (2017) 9. doi: 10.1126/scitranslmed.aah5360, PMID: 28931657 PMC5842431

[85] Feng Z Qi S Zhang Y Qi Z Yan L Zhou J . Ly6g+ Neutrophil-Derived Mir-223 Inhibits the Nlrp3 Inflammasome in Mitochondrial Damp-Induced Acute Lung Injury. Cell Death Dis. (2017) 8:e3170. doi: 10.1038/cddis.2017.549, PMID: 29144508 PMC5775410

[86] Zeng Z Qin Y He X Tan Z . Microrna-223/Ne Signaling Pathway Inhibits Lipopolysaccharide-Induced Acute Lung Injury by Regulating Neutrophil Extracellular Traps. Mediators Inflammation. (2026) 2026:1621608. doi: 10.1155/mi/1621608, PMID: 41532006 PMC12794271

[87] Chen B Han J Chen S Xie R Yang J Zhou T . Microlet-7b Regulates Neutrophil Function and Dampens Neutrophilic Inflammation by Suppressing the Canonical Tlr4/Nf-κb Pathway. Front Immunol. (2021) 12:653344. doi: 10.3389/fimmu.2021.653344, PMID: 33868293 PMC8044834

[88] Smith S Wu PW Seo JJ Fernando T Jin M Contreras J . Il-16/Mir-125a Axis Controls Neutrophil Recruitment in Pristane-Induced Lung Inflammation. *JCI insight* (2018) 3(15). (2018) Epub 2018/08/10. doi: 10.1172/jci.insight.120798, PMID: PMC6129123 30089723

[89] Lai X Guo Y Chen M Wei Y Yi W Shi Y . Caveolin1: Its Roles in Normal and Cancer Stem Cells. J Cancer Res Clin Oncol. (2021) 147:3459–75. doi: 10.1007/s00432-021-03793-2, PMID: 34498146 PMC11802073

[90] Rafati F Ghorbani Z Manoochehrabadi T Khosravi F Majidi J Eskandari F . Smart Integrated Biomaterial Systems for Precision and Optimized Delivery of Mscs and Their Exosomes: Transforming Wound Healing and Organ Regeneration. Regenerative Ther. (2026) 31:101045. doi: 10.1016/j.reth.2025.101045, PMID: 41399764 PMC12702032

[91] Zheng XL Gu WJ Zhang F Zhao FZ Li LZ Huang HY . Exosomal Mir-127-5p from Bmscs Alleviated Sepsis-Related Acute Lung Injury by Inhibiting Neutrophil Extracellular Trap Formation. Int Immunopharmacol. (2023) 123:110759. doi: 10.1016/j.intimp.2023.110759, PMID: 37552907

[92] Swaminathan AC Todd JL Palmer SM . Advances in Human Lung Transplantation. Annu Rev Med. (2021) 72:135–49. doi: 10.1146/annurev-med-080119-103200, PMID: 33113336

[93] Wu J Gao P Yang C Zhuang F Luo Y Wen F . Targeting Mitochondrial Complex I of Cd177(+) Neutrophils Alleviates Lung Ischemia-Reperfusion Injury. Cell Rep Med. (2025) 6:102140. doi: 10.1016/j.xcrm.2025.102140, PMID: 40398393 PMC12147905

[94] Sayah DM Mallavia B Liu F Ortiz-Muñoz G Caudrillier A DerHovanessian A . Neutrophil Extracellular Traps Are Pathogenic in Primary Graft Dysfunction after Lung Transplantation. Am J Respir Crit Care Med. (2015) 191:455–63. doi: 10.1164/rccm.201406-1086OC, PMID: 25485813 PMC4351593

[95] Li J Wei L Han Z Chen Z Zhang Q . Long Non-Coding Rna X-Inactive Specific Transcript Silencing Ameliorates Primary Graft Dysfunction Following Lung Transplantation through Microrna-21-Dependent Mechanism. EBioMedicine. (2020) 52:102600. doi: 10.1016/j.ebiom.2019.102600, PMID: 31981974 PMC6976928

[96] Lambrecht BN Ahmed E Hammad H . The Immunology of Asthma. Nat Immunol. (2025) 26:1233–45. doi: 10.1038/s41590-025-02212-9, PMID: 40730897

[97] Russell RJ Brightling C . Pathogenesis of Asthma: Implications for Precision Medicine. Clin Sci (London England: 1979). (2017) 131:1723–35. doi: 10.1042/cs20160253, PMID: 28667070

[98] Hammad H Ahmed E Lambrecht BN . Immunotherapy for Asthma. Cell Mol Immunol. (2025) 22:1521–32. doi: 10.1038/s41423-025-01357-9, PMID: 41145900 PMC12660796

[99] Lachowicz-Scroggins ME Dunican EM Charbit AR Raymond W Looney MR Peters MC . Neutrophil Extracellular Traps, and Inflammasome Activation in Severe Asthma. Am J Respir Crit Care Med. (2019) 199:1076–85. doi: 10.1164/rccm.201810-1869OC, PMID: 30888839 PMC6515873

[100] Crisford H Sapey E Rogers GB Taylor S Nagakumar P Lokwani R . Neutrophils in Asthma: The Good, the Bad and the Bacteria. Thorax. (2021) 76:835–44. doi: 10.1136/thoraxjnl-2020-215986, PMID: 33632765 PMC8311087

[101] Tsai CH Lai AC Lin YC Chi PY Chen YC Yang YH . Neutrophil Extracellular Trap Production and Ccl4l2 Expression Influence Corticosteroid Response in Asthma. Sci Trans Med. (2023) 15:eadf3843. doi: 10.1126/scitranslmed.adf3843, PMID: 37285400

[102] Zuyderduyn S Sukkar MB Fust A Dhaliwal S Burgess JK . Treating Asthma Means Treating Airway Smooth Muscle Cells. Eur Respir J. (2008) 32:265–74. doi: 10.1183/09031936.00051407, PMID: 18669785

[103] Maes T Cobos FA Schleich F Sorbello V Henket M De Preter K . Asthma Inflammatory Phenotypes Show Differential Microrna Expression in Sputum. J Allergy Clin Immunol. (2016) 137:1433–46. doi: 10.1016/j.jaci.2016.02.018, PMID: 27155035

[104] Kim JY Stevens P Karpurapu M Lee H Englert JA Yan P . Targeting Etosis by Mir-155 Inhibition Mitigates Mixed Granulocytic Asthmatic Lung Inflammation. Front Immunol. (2022) 13:943554. doi: 10.3389/fimmu.2022.943554, PMID: 35958610 PMC9360579

[105] Vargas A Roux-Dalvai F Droit A Lavoie JP . Neutrophil-Derived Exosomes: A New Mechanism Contributing to Airway Smooth Muscle Remodeling. Am J Respir Cell Mol Biol. (2016) 55:450–61. doi: 10.1165/rcmb.2016-0033OC, PMID: 27105177

[106] Vargas A Mainguy-Seers S Boivin R Lavoie JP . Low Levels of Microrna-21 in Neutrophil-Derived Exosomes May Contribute to Airway Smooth Muscle Hyperproliferation in Horses with Severe Asthma. Am J veterinary Res. (2024) 85. doi: 10.2460/ajvr.23.11.0267, PMID: 38382196

[107] He L Qiang R Li W . The Mir-3164/Pad4 Axis Regulates Netosis to Prevent Airway Inflammation and Remodeling through the Tlr2/Nf-κb Signaling Pathway. Eur J Med Res. (2025) 30:947. doi: 10.1186/s40001-025-03175-1, PMID: 41068990 PMC12512812

[108] Sim S Lee DH Kim KS Park HJ Kim YK Choi Y . Micrococcus Luteus-Derived Extracellular Vesicles Attenuate Neutrophilic Asthma by Regulating Mirnas in Airway Epithelial Cells. Exp Mol Med. (2023) 55:196–204. doi: 10.1038/s12276-022-00910-0, PMID: 36639716 PMC9898544

[109] Xu W Wang Y Ma Y Yang J . Mir-223 Plays a Protecting Role in Neutrophilic Asthmatic Mice through the Inhibition of Nlrp3 Inflammasome. Respir Res. (2020) 21:116. doi: 10.1186/s12931-020-01374-4, PMID: 32423405 PMC7236263

[110] Wynn TA . Integrating Mechanisms of Pulmonary Fibrosis. J Exp Med. (2011) 208:1339–50. doi: 10.1084/jem.20110551, PMID: 21727191 PMC3136685

[111] Fabre A Marchal-Sommé J Marchand-Adam S Quesnel C Borie R Dehoux M . Modulation of Bleomycin-Induced Lung Fibrosis by Serotonin Receptor Antagonists in Mice. Eur Respir J. (2008) 32:426–36. doi: 10.1183/09031936.00126907, PMID: 18321937

[112] Li C Lu Y Du S Li S Zhang Y Liu F . Dioscin Exerts Protective Effects against Crystalline Silica-Induced Pulmonary Fibrosis in Mice. Theranostics. (2017) 7:4255–75. doi: 10.7150/thno.20270, PMID: 29158824 PMC5695011

[113] Hettiarachchi SU Li YH Roy J Zhang F Puchulu-Campanella E Lindeman SD . Targeted Inhibition of Pi3 Kinase/Mtor Specifically in Fibrotic Lung Fibroblasts Suppresses Pulmonary Fibrosis in Experimental Models. Sci Trans Med. (2020) 12. doi: 10.1126/scitranslmed.aay3724, PMID: 33115948

[114] Liu P Miao K Zhang L Mou Y Xu Y Xiong W . Curdione Ameliorates Bleomycin-Induced Pulmonary Fibrosis by Repressing Tgf-β-Induced Fibroblast to Myofibroblast Differentiation. Respir Res. (2020) 21:58. doi: 10.1186/s12931-020-1300-y, PMID: 32075634 PMC7031930

[115] Crowley LE Stockley RA Thickett DR Dosanjh D Scott A Parekh D . Neutrophil Dynamics in Pulmonary Fibrosis: Pathophysiological and Therapeutic Perspectives. Eur Respir review: an Off J Eur Respir Soc. (2024) 33. doi: 10.1183/16000617.0139-2024, PMID: 39603661 PMC11600124

[116] Zhang S Jia X Zhang Q Zhang L Yang J Hu C . Neutrophil Extracellular Traps Activate Lung Fibroblast to Induce Polymyositis-Related Interstitial Lung Diseases Via Tlr9-Mir-7-Smad2 Pathway. J Cell Mol Med. (2020) 24:1658–69. doi: 10.1111/jcmm.14858, PMID: 31821687 PMC6991674

[117] Tang LB Peng YL Chen J Li JT Zheng MM Wu L . Rechallenge with Immune-Checkpoint Inhibitors in Patients with Advanced-Stage Lung Cancer. Nat Rev Clin Oncol. (2025) 22:546–65. doi: 10.1038/s41571-025-01029-7, PMID: 40490476

[118] Wu KL Tsai YM Lien CT Kuo PL Hung AJ . The Roles of Microrna in Lung Cancer. Int J Mol Sci. (2019) 20. doi: 10.3390/ijms20071611, PMID: 30935143 PMC6480472

[119] Morgensztern D Ng SH Gao F Govindan R . Trends in Stage Distribution for Patients with Non-Small Cell Lung Cancer: A National Cancer Database Survey. J Thorac Oncol Off Publ Int Assoc Study Lung Cancer. (2010) 5:29–33. doi: 10.1097/JTO.0b013e3181c5920c, PMID: 19952801

[120] Wang J Zhang M Cui Z Qu J Chang R . Bidirectional Role of Neutrophils in Lung Cancer: Mechanisms and Therapeutic Implications. Crit Rev oncology/hematol. (2025) 218:105087. doi: 10.1016/j.critrevonc.2025.105087, PMID: 41421459

[121] Hu S Yan C Tian Y Sun W . Neutrophils in Non-Small Cell Lung Cancer and Immunotherapy with Pd-1/Pd-L1 Inhibitors. J Trans Med. (2025) 23:1313. doi: 10.1186/s12967-025-07084-z, PMID: 41254689 PMC12625643

[122] Fridlender ZG Sun J Kim S Kapoor V Cheng G Ling L . Polarization of Tumor-Associated Neutrophil Phenotype by Tgf-Beta: “N1” Versus “N2” Tan. Cancer Cell. (2009) 16:183–94. doi: 10.1016/j.ccr.2009.06.017, PMID: 19732719 PMC2754404

[123] Kargl J Busch SE Yang GH Kim KH Hanke ML Metz HE . Neutrophils Dominate the Immune Cell Composition in Non-Small Cell Lung Cancer. Nat Commun. (2017) 8:14381. doi: 10.1038/ncomms14381, PMID: 28146145 PMC5296654

[124] Eruslanov EB Bhojnagarwala PS Quatromoni JG Stephen TL Ranganathan A Deshpande C . Tumor-Associated Neutrophils Stimulate T Cell Responses in Early-Stage Human Lung Cancer. J Clin Invest. (2014) 124:5466–80. doi: 10.1172/jci77053, PMID: 25384214 PMC4348966

[125] Horvath L Puschmann C Scheiber A Martowicz A Sturm G Trajanoski Z . Beyond Binary: Bridging Neutrophil Diversity to New Therapeutic Approaches in Nsclc. Trends Cancer. (2024) 10:457–74. doi: 10.1016/j.trecan.2024.01.010, PMID: 38360439

[126] Ancey PB Contat C Boivin G Sabatino S Pascual J Zangger N . Glut1 Expression in Tumor-Associated Neutrophils Promotes Lung Cancer Growth and Resistance to Radiotherapy. Cancer Res. (2021) 81:2345–57. doi: 10.1158/0008-5472.Can-20-2870, PMID: 33753374 PMC8137580

[127] Anderson R Blidner AG Rapoport BL . Frontiers in Pharmacology: Review Manuscript Targeting of the Neutrophil as an Adjunctive Strategy in Non-Small Cell Lung Cancer. Front Pharmacol. (2021) 12:676399. doi: 10.3389/fphar.2021.676399, PMID: 34168563 PMC8218630

[128] Zhang X Huang X Zhang X Lai L Zhu B Lin P . The Mir-941/Foxn4/Tgf-β Feedback Loop Induces N2 Polarization of Neutrophils and Enhances Tumor Progression of Lung Adenocarcinoma. Front Immunol. (2025) 16:1561081. doi: 10.3389/fimmu.2025.1561081, PMID: 40352924 PMC12061992

[129] Fortunato O Borzi C Milione M Centonze G Conte D Boeri M . Circulating Mir-320a Promotes Immunosuppressive Macrophages M2 Phenotype Associated with Lung Cancer Risk. Int J Cancer. (2019) 144:2746–61. doi: 10.1002/ijc.31988, PMID: 30426475 PMC6590261

[130] Ma J Li N Lin Y Gupta C Jiang F . Circulating Neutrophil Micrornas as Biomarkers for the Detection of Lung Cancer. Biomarkers Cancer. (2016) 8:1–7. doi: 10.4137/bic.S37333, PMID: 26823654 PMC4725606

[131] Manzari MT Shamay Y Kiguchi H Rosen N Scaltriti M Heller DA . Targeted Drug Delivery Strategies for Precision Medicines. Nat Rev Materials. (2021) 6:351–70. doi: 10.1038/s41578-020-00269-6, PMID: 34950512 PMC8691416

[132] Chu D Dong X Shi X Zhang C Wang Z . Neutrophil-Based Drug Delivery Systems. Advanced materials (Deerfield Beach Fla). (2018) 30:e1706245. doi: 10.1002/adma.201706245, PMID: 29577477 PMC6161715

[133] Yuan S Hu Q . Convergence of Nanomedicine and Neutrophils for Drug Delivery. Bioactive materials. (2024) 35:150–66. doi: 10.1016/j.bioactmat.2024.01.022, PMID: 38318228 PMC10839777

[134] Li Y Zhang X Li Y Sun L Hu N Lui S . Neutrophil-Based Delivery Platforms: From Natural Mechanisms to Engineered Therapeutics. Theranostics. (2026) 16:123–55. doi: 10.7150/thno.117363, PMID: 41328335 PMC12665108

[135] Zeng C Duan F Hu J Luo B Huang B Lou X . Nlrp3 Inflammasome-Mediated Pyroptosis Contributes to the Pathogenesis of Non-Ischemic Dilated Cardiomyopathy. Redox Biol. (2020) 34:101523. doi: 10.1016/j.redox.2020.101523, PMID: 32273259 PMC7327979

[136] Ma C Liu K Wang F Fei X Niu C Li T . Neutrophil Membrane-Engineered Panax Ginseng Root-Derived Exosomes Loaded Mirna 182-5p Targets Nox4/Drp-1/Nlrp3 Signal Pathway to Alleviate Acute Lung Injury in Sepsis: Experimental Studies. Int J Surg (London England). (2024) 110:72–86. doi: 10.1097/js9.0000000000000789, PMID: 37737899 PMC10793765

[137] Wang G Ma X Huang W Wang S Lou A Wang J . Macrophage Biomimetic Nanoparticle-Targeted Functional Extracellular Vesicle Micro-Rnas Revealed Via Multiomics Analysis Alleviate Sepsis-Induced Acute Lung Injury. J nanobiotechnol. (2024) 22:362. doi: 10.1186/s12951-024-02597-z, PMID: 38910259 PMC11194988

[138] Das S Kaminski TW Schlegel BT Bain W Hu S Patel A . Neutrophils and Galectin-3 Defend Mice from Lethal Bacterial Infection and Humans from Acute Respiratory Failure. Nat Commun. (2024) 15:4724. doi: 10.1038/s41467-024-48796-y, PMID: 38830855 PMC11148175

[139] Gu M Pang Z . Luteolin Inhibits Inflammation and M1 Macrophage Polarization in the Treatment of Pseudomonas Aeruginosa-Induced Acute Pneumonia through Suppressing Egfr/Pi3k/Akt/Nf-κb and Egfr/Erk/Ap-1 Signaling Pathways. Phytomedicine: Int J phytotherapy phytopharmacol. (2025) 141:156663. doi: 10.1016/j.phymed.2025.156663, PMID: 40133026

[140] Tay HL Kaiko GE Plank M Li J Maltby S Essilfie AT . Antagonism of Mir-328 Increases the Antimicrobial Function of Macrophages and Neutrophils and Rapid Clearance of Non-Typeable Hemophilus Influenzae (Nthi) from Infected Lung. PloS Pathog. (2015) 11:e1004549. doi: 10.1371/journal.ppat.1004549, PMID: 25894560 PMC4404141

[141] Schmidt B Roberts RS Davis P Doyle LW Barrington KJ Ohlsson A . Caffeine Therapy for Apnea of Prematurity. New Engl J Med. (2006) 354:2112–21. doi: 10.1056/NEJMoa054065, PMID: 16707748

[142] Li X He S Li R Zhou X Zhang S Yu M . Pseudomonas Aeruginosa Infection Augments Inflammation through Mir-301b Repression of C-Myb-Mediated Immune Activation and Infiltration. Nat Microbiol. (2016) 1:16132. doi: 10.1038/nmicrobiol.2016.132, PMID: 27670114 PMC5061341

[143] Jansson AH Eriksson C Wang X . Effects of Budesonide and N-Acetylcysteine on Acute Lung Hyperinflation, Inflammation and Injury in Rats. Vasc Pharmacol. (2005) 43:101–11. doi: 10.1016/j.vph.2005.03.006, PMID: 15967733

[144] Wei X Feng L Chen F . Acetylcysteine Combined with Budesonide Nebulized Inhalation for the Treatment of Pneumonia in Children: A Meta-Analysis. Eur J Clin Pharmacol. (2026) 82:33. doi: 10.1007/s00228-025-03956-x, PMID: 41546714

[145] Chen J Zhu Y Zheng C Zhao W Liu Q . Clinical Efficacy of Budesonide Combined with Acetylcysteine in the Treatment of Mycoplasma Pneumonia Infection. Immunity Inflammation Dis. (2023) 11:e1068. doi: 10.1002/iid3.1068, PMID: 38018572 PMC10664398

[146] Li Y Yu H Lv M Li Q Zou K Lv S . Combination Therapy with Budesonide and N-Acetylcysteine Ameliorates Lps-Induced Ali by Attenuating Neutrophil Recruitment through the Mir-196b-5p/Socs3 Molecular Axis. BMC pulmonary Med. (2022) 22:388. doi: 10.1186/s12890-022-02185-7, PMID: 36289489 PMC9608916

[147] Stevens RM Ervin J Nezzer J Nieves Y Guedes K Burges R . Randomized, Double-Blind, Placebo-Controlled Trial of Intraarticular Trans-Capsaicin for Pain Associated with Osteoarthritis of the Knee. Arthritis Rheumatol (Hoboken NJ). (2019) 71:1524–33. doi: 10.1002/art.40894, PMID: 30888737 PMC6772016

[148] Zebda D Jiang ZY Gibson MM Pham C Ahmadi S Floren S . Double-Blinded Randomized Prospective Trial of Intranasal Capsaicin Treatment for Nonallergic Rhinitis. Int Forum Allergy rhinology. (2021) 11:24–30. doi: 10.1002/alr.22637, PMID: 33045140

[149] Adel RM Helal H Ahmed Fouad M Sobhy Abd-Elhalem S . Regulation of Mirna-155-5p Ameliorates Netosis in Pulmonary Fibrosis Rat Model Via Inhibiting Its Target Cytokines Il-1β, Tnf-α and Tgf-β1. Int Immunopharmacol. (2024) 127:111456. doi: 10.1016/j.intimp.2023.111456, PMID: 38159555

[150] Garreau M Weidner J Hamilton R Kolosionek E Toki N Stavenhagen K . Chemical Modification Patterns for Microrna Therapeutic Mimics: A Structure-Activity Relationship (Sar) Case-Study on Mir-200c. Nucleic Acids Res. (2024) 52:2792–807. doi: 10.1093/nar/gkae141, PMID: 38421619 PMC11014349

[151] Valizadeh-Otaghsara M Haji Molla Hoseini M Karima S Zali H Khoshnevisan K Mohammadi-Yeganeh S . Mirna Delivery Systems in Cancer Stem Cell Therapy: Exosomes Versus Chitosan Nanoparticles. Stem Cell Res Ther. (2025) 16:576. doi: 10.1186/s13287-025-04667-x, PMID: 41126279 PMC12542416

[152] Lee TJ Yuan X Kerr K Yoo JY Kim DH Kaur B . Strategies to Modulate Microrna Functions for the Treatment of Cancer or Organ Injury. Pharmacol Rev. (2020) 72:639–67. doi: 10.1124/pr.119.019026, PMID: 32554488 PMC7300323

[153] Diener C Keller A Meese E . Emerging Concepts of Mirna Therapeutics: From Cells to Clinic. Trends Genet. (2022) 38:613–26. doi: 10.1016/j.tig.2022.02.006, PMID: 35303998

[154] Dogra P Shinglot V Ruiz-Ramírez J Cave J Butner JD Schiavone C . Translational Modeling-Based Evidence for Enhanced Efficacy of Standard-of-Care Drugs in Combination with Anti-Microrna-155 in Non-Small-Cell Lung Cancer. Mol Cancer. (2024) 23:156. doi: 10.1186/s12943-024-02060-5, PMID: 39095771 PMC11295620

[155] Shukuya T Ghai V Amann JM Okimoto T Shilo K Kim TK . Circulating Micrornas and Extracellular Vesicle-Containing Micrornas as Response Biomarkers of Anti-Programmed Cell Death Protein 1 or Programmed Death-Ligand 1 Therapy in Nsclc. J Thorac Oncol Off Publ Int Assoc Study Lung Cancer. (2020) 15:1773–81. doi: 10.1016/j.jtho.2020.05.022, PMID: 32565389 PMC7641981

[156] Tay Y Rinn J Pandolfi PP . The Multilayered Complexity of Cerna Crosstalk and Competition. Nature. (2014) 505:344–52. doi: 10.1038/nature12986, PMID: 24429633 PMC4113481

[157] Wei L Li J Han Z Chen Z Zhang Q . Silencing of Lncrna Malat1 Prevents Inflammatory Injury after Lung Transplant Ischemia-Reperfusion by Downregulation of Il-8 Via P300. Mol Ther Nucleic Acids. (2019) 18:285–97. doi: 10.1016/j.omtn.2019.05.009, PMID: 31604167 PMC6796730

[158] Li J Han Z Zhu Z Wei L . Lncrna H19 Aggravates Primary Graft Dysfunction after Lung Transplantation Via Klf5-Mediated Activation of Ccl28. Am J transplantation: Off J Am Soc Transplant Am Soc Transplant Surgeons. (2023) 23:1536–50. doi: 10.1016/j.ajt.2023.06.015, PMID: 37394140

[159] Zhang XY Chen ZC Li N Wang ZH Guo YL Tian CJ . Exosomal Transfer of Activated Neutrophil-Derived Lncrna Crnde Promotes Proliferation and Migration of Airway Smooth Muscle Cells in Asthma. Hum Mol Genet. (2022) 31:638–50. doi: 10.1093/hmg/ddab283, PMID: 34590683

[160] Wang Y Liu F Chen L Fang C Li S Yuan S . Neutrophil Extracellular Traps (Nets) Promote Non-Small Cell Lung Cancer Metastasis by Suppressing Lncrna Mir503hg to Activate the Nf-κb/Nlrp3 Inflammasome Pathway. Front Immunol. (2022) 13:867516. doi: 10.3389/fimmu.2022.867516, PMID: 35707534 PMC9190762

[161] Ye X Fang C Hong W Qian X Yu B Zhou B . Lncrna Mir503hg Regulates Nets-Mediated Nlrp3 Inflammasome Activation and Nsclc Metastasis by Enhancing the Ubiquitination of C/Ebpβ. Clin Trans Med. (2025) 15:e70342. doi: 10.1002/ctm2.70342, PMID: 40490947 PMC12148952

[162] Feng Y Liu G Li H Cheng L . Target Neutrophil Heterogeneity and Plasticity in Cancer. J Hematol Oncol. (2025) 18:79. doi: 10.1186/s13045-025-01731-0, PMID: 40797279 PMC12345094

[163] Garley M Nowak K Jabłońska E . Neutrophil Micrornas. Biol Rev Cambridge Philos Soc. (2024) 99:864–77. doi: 10.1111/brv.13048, PMID: 38148491

[164] Nemeth K Bayraktar R Ferracin M Calin GA . Non-Coding Rnas in Disease: From Mechanisms to Therapeutics. Nat Rev Genet. (2024) 25:211–32. doi: 10.1038/s41576-023-00662-1, PMID: 37968332

